# Rare heterozygous *GDF6* variants in patients with renal anomalies

**DOI:** 10.1038/s41431-020-0678-9

**Published:** 2020-07-31

**Authors:** Helge Martens, Imke Hennies, Maike Getwan, Anne Christians, Anna-Carina Weiss, Frank Brand, Ann Christin Gjerstad, Arne Christians, Zoran Gucev, Robert Geffers, Tomáš Seeman, Andreas Kispert, Velibor Tasic, Anna Bjerre, Soeren S. Lienkamp, Dieter Haffner, Ruthild G. Weber

**Affiliations:** 1 Department of Human Genetics, Hannover Medical School, 30625 Hannover, Germany; 2 Department of Pediatric Kidney, Liver and Metabolic Diseases, Hannover Medical School, 30625 Hannover, Germany; 3 Department of Medicine, Renal Division, University Medical Center Freiburg, Faculty of Medicine, University of Freiburg, 79110 Freiburg, Germany; 4 Institute of Anatomy and Zurich Center for Integrative Human Physiology (ZIHP), University of Zurich, 8057 Zurich, Switzerland; 5 Institute of Molecular Biology, Hannover Medical School, 30625 Hannover, Germany; 6 Division of Paediatric and Adolescent Medicine, Oslo University Hospital, 0424 Oslo, Norway; 7 Department of Neuropathology, Institute of Pathology, Hannover Medical School, 30625 Hannover, Germany; 8 Medical Faculty Skopje, University Children’s Hospital, 1000 Skopje, North Macedonia; 9 Genome Analytics Research Group, Helmholtz Centre for Infection Research, 38124 Braunschweig, Germany; 10 Department of Paediatrics and Transplantation Center, University Hospital Motol, Second Faculty of Medicine, Charles University, 150 06 Prague, Czech Republic

**Keywords:** Genetics research, Mutation, Development, Medical genetics

## Abstract

Although over 50 genes are known to cause renal malformation if mutated, the underlying genetic basis, most easily identified in syndromic cases, remains unsolved in most patients. In search of novel causative genes, whole-exome sequencing in a patient with renal, i.e., crossed fused renal ectopia, and extrarenal, i.e., skeletal, eye, and ear, malformations yielded a rare heterozygous variant in the *GDF6* gene encoding growth differentiation factor 6, a member of the BMP family of ligands. Previously, *GDF6* variants were reported to cause pleiotropic defects including skeletal, e.g., vertebral, carpal, tarsal fusions, and ocular, e.g., microphthalmia and coloboma, phenotypes. To assess the role of *GDF6* in the pathogenesis of renal malformation, we performed targeted sequencing in 193 further patients identifying rare *GDF6* variants in two cases with kidney hypodysplasia and extrarenal manifestations. During development, *gdf6* was expressed in the pronephric tubule of *Xenopus laevis*, and *Gdf6* expression was observed in the ureteric tree of the murine kidney by RNA in situ hybridization. CRISPR/Cas9-derived knockout of *Gdf6* attenuated migration of murine IMCD3 cells, an effect rescued by expression of wild-type but not mutant *GDF6*, indicating affected variant function regarding a fundamental developmental process. Knockdown of *gdf6* in *Xenopus laevis* resulted in impaired pronephros development. Altogether, we identified rare heterozygous *GDF6* variants in 1.6% of all renal anomaly patients and 5.4% of renal anomaly patients additionally manifesting skeletal, ocular, or auricular abnormalities, adding renal hypodysplasia and fusion to the phenotype spectrum of *GDF6* variant carriers and suggesting an involvement of GDF6 in nephrogenesis.

## Introduction

Structural defects of the kidney range from renal agenesis, hypoplasia, and dysplasia to duplication and fusion phenotypes, such as horseshoe kidneys and crossed fused renal ectopia. The latter is a rare form of renal anomaly where two fused kidneys come to lie on the same side of the spine, each with their own ureter, one of which crossing the midline to enter the bladder on the contralateral side. As other renal anomalies are also frequently associated with malformations of the urinary tract, such as ureteropelvic or ureterovesical junction obstruction with hydroureter, or vesicoureteral reflux (VUR), the term congenital anomalies of the kidney and urinary tract (CAKUT) has been coined to subsume these abnormalities. Taken together, CAKUT phenotypes account for 15–30% of all prenatally detected congenital malformations [1], and cause around 40% of cases with end-stage kidney disease in children and adolescents [2], thus representing a significant health burden. In around 85% of patients, CAKUT occur sporadically, while in the remaining 15% of cases familial occurrence is observed. CAKUT may occur in isolation or be part of a mild or complex syndromal disease. Since over 500 syndromes have been associated with CAKUT [3], it is not surprising that one-third of patients are additionally affected by extrarenal manifestations [4], and that around 20% of patients may have a genetic disorder that is not detected based on standard clinical evaluation [5]. Although over 50 genes are known to cause CAKUT in humans if mutated [6, 7], <20% of CAKUT manifestations can be explained by aberrations in these genes [8, 9], indicating a high genetic heterogeneity underlying these defects and making clear the need to identify new genes associated with renal development and malformation. However, the identification of causative genetic variants in cohorts of sporadic CAKUT patients and CAKUT families is hampered by variable expressivity, meaning that individuals harboring the same variant may have very different phenotypes within the broad CAKUT spectrum, and by incomplete penetrance implying that variant carriers may exist that are not affected by a CAKUT phenotype at all.

In the last few years, whole-exome sequencing (WES) using next generation sequencing (NGS) techniques was successfully applied to the study of germline variation underlying human CAKUT [10]. Thereby, novel CAKUT-associated genes were identified e.g., by using a linkage-based strategy in large CAKUT families [11, 12], a double hit-based strategy in smaller CAKUT families [13], an overlapping strategy in a cohort of patients with similar phenotypes [14], and a trio-based de novo strategy in patients with sporadic CAKUT [15, 16]. In addition, WES has improved the diagnostic yield of genetic CAKUT causes, particularly in syndromic cases [9].

Here, in an effort to identify new genes associated with renal malformation in humans, we used a WES approach to determine the genetic variation underlying renal anomalies in an index patient with syndromic CAKUT. By a targeted mutational screen in a cohort of 193 further patients with renal anomalies, expression analyses, and functional studies in a cellular system modified by CRISPR/Cas9 genome engineering and an animal model, we suggest that the candidate gene identified in the index patient, *GDF6*, plays a role in kidney development and malformation.

## Subjects and methods

### Patients

This study was conducted in accordance with the Declaration of Helsinki and approved by the Ethics Boards of Hannover Medical School, Hannover, Germany, Oslo University Hospital, Oslo, Norway, and University Children’s Hospital, Skopje, North Macedonia. Each family provided informed consent for participation in the study. A total of 194 patients with renal anomalies comprising 122 males and 72 females with a mean age of 10 years (range 1–35 years) were analyzed. Renal phenotypes of all 194 patients are listed in Supplementary Table 1. Rare heterozygous *GDF6* variants predicted to be disease causing were detected in families F006, H435, and N038.

#### Family F006

**Fig. 1:**
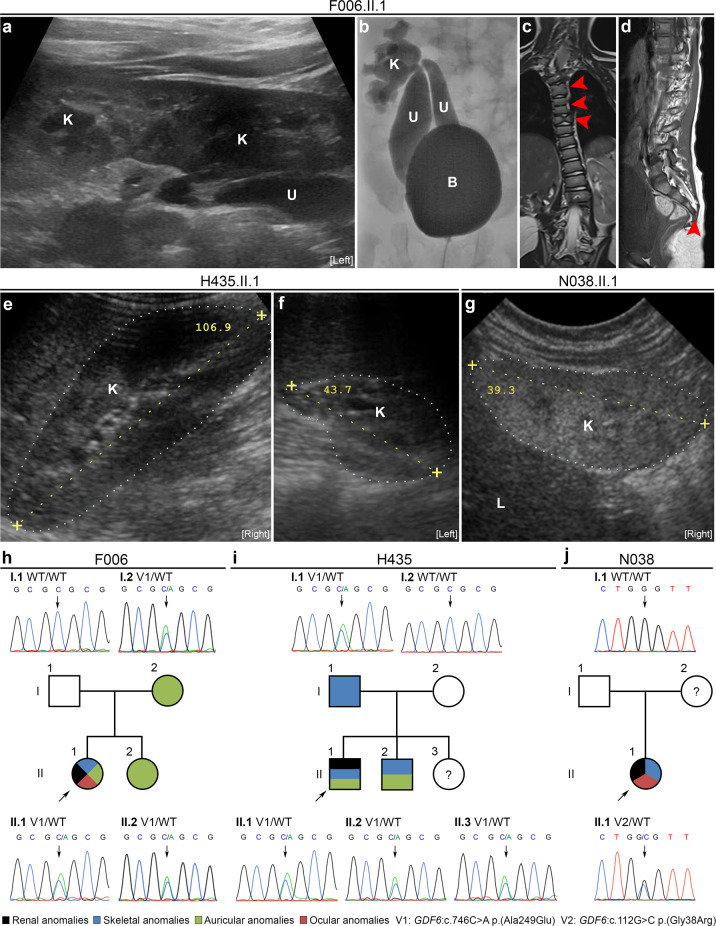
WES and targeted sequencing identified heterozygous rare *GDF6* variants in three of 194 patients with renal anomalies (1.6%), and in three of 56 patients with renal plus skeletal, ocular or auricular anomalies (5.4%). **a** Renal ultrasound of index patient F006.II.1 was notable for left-sided crossed fused kidney (K) ectopia with megaureters (U). **b** Voiding cystourethrography of patient F006.II.1 revealed grade-IV vesicoureteral reflux into two megaureters (U) with orthotopic ostia in the bladder (B) and connected to the superior or inferior dilated kidney (K) pelvis of the fused kidneys. **c**, **d** MRI of the spine of patient F006.II.1 showing malformations of cervical and thoracic vertebral bodies, e.g., fused and butterfly vertebrae (arrows) (**c**) and missing fifth sacral vertebral body and coccyx (arrow) (**d**). **e**, **f** Renal ultrasound of patient H435.II.1 at the age of 11 years was unremarkable on the right side (**e**), while the left kidney was hypodysplastic (**f**) and nonfunctional on DMSA scan. **g** Renal ultrasound of patient N038.II.1 at age 6 months showing renal hypodysplasia, as indicated by reduced size, hyperechogenicity, and reduced corticomedullary differentiation of the right kidney depicted. The left kidney was also hypodysplastic (not shown), and the patient required kidney transplantation at 5 years of age. **h**–**j** Pedigrees of families F006 (**h**), H435 (**i**), and N038 (**j**) with colored shading indicating phenotypical overlap with respect to renal, skeletal, auricular, and ocular anomalies in individuals with rare *GDF6* variants. The *GDF6* mutational status (V1: c.746C>A p.(Ala249Glu), V2: c.112G>C p.(Gly38Arg), WT: wild-type) is indicated, and corresponding electropherograms are shown for all analyzed family members (no DNA sample was available from individual N038.I.2). Clinical and radiological information was not available from individuals H435.II.3 (1 year of age) or N038.I.2. L, liver; the kidneys are marked by dotted lines (**e**–**g**).

The index patient, F006.II.1, born as the second daughter of non-consanguineous German parents is now 3 years old. After birth, renal ultrasound was notable for left-sided crossed fused renal ectopia (Fig. 1a). Voiding cystourethrography revealed two megaureters, both with orthotopic ostia in the bladder, one connected to the superior pelvis, and the other to the inferior pelvis of the left-sided fused kidneys (Fig. 1b), and grade-IV VUR in both ureters. Recurrent urinary tract infections were diagnosed. The patient also showed a left-convex torsion scoliosis, malformations of multiple vertebral bodies of the cervical and thoracic spine including butterfly and fused vertebrae, and a missing fifth sacral vertebral body and coccyx (Fig. 1c, d). A tethered cord was diagnosed because of a low standing *conus medullaris*, and detethering surgery was performed. The patient also presented with anal atresia and a rectovestibular fistula, which was surgically corrected. By echocardiography, two small muscular ventricular septal defects and a patent *foramen ovale* were diagnosed. Ophthalmologic examination revealed anisometropia with hyperopia, astigmatism, amblyopia, suspected microphthalmia, corneal opacities, and a best-corrected visual acuity of 0.16 in the left eye and of 1.0 in the right eye. In addition, the patient presented with a left-sided auricle dysplasia and aplasia of the external auditory canal, while the cochleae and the semicircular canals were unremarkable on both sides according to cranial MRI. Neurological examination was normal and no developmental or intellectual deficits were observed. Her 6-year-old sister and 36-year-old mother presented with right-sided preauricular pits, while renal ultrasound examinations revealed no abnormalities. Her father was clinically unremarkable and renal sonography was normal.

#### Family H435

Twelve-year-old patient H435.II.1 was born as the second son of non-consanguineous Macedonian parents. After birth, the boy presented with a right-sided normal kidney and a left-sided hypodysplastic kidney, a diagnosis confirmed by ultrasound at age 11 years (Fig. 1e, f). The left kidney could not be visualized by a radionuclide scan using DMSA indicating that it is not functional. In addition, the patient showed a mild torsion scoliosis. The philtrum appeared shorter than usual, a high-arched palate and malocclusion due to prognathism were noted. His ears were of normal size but had a slight lop deformity. Except for the presence of a short frenulum, external genitalia were unremarkable. No developmental or intellectual deficits were noted. His 15-year-old brother also had high-arched palate and lop ears, whereby particularly the right ear was smaller and showed a poorly developed antihelix. A triangular-shaped chin was observed. Neurological examination was normal and no developmental or intellectual deficits were diagnosed. His 1-year-old sister was clinically unremarkable, but renal ultrasound or other examinations were not performed. His father presented with short stature. Renal sonography of both parents was normal.

#### Family N038

Fourteen-year-old patient N038.II.1 is the only daughter of an African mother and a Caucasian father. She was prenatally diagnosed with oligohydramnios and small kidneys. Renal ultrasound at age 6 months showed bilateral renal hypodysplasia as indicated by reduced size, hyperechogenicity, and reduced corticomedullary differentiation of the kidneys (right kidney shown in Fig. 1g). After birth, she additionally presented with macrocephaly, high-arched palate, and short narrow palpebral fissures. She required kidney transplantation at the age of 5 years and was re-transplanted at 9 years of age due to chronic rejection and noncompliance with drug treatment. At 14 years, she has poor kidney function and several comorbidities such as obesity and hypertension.

### Animals

Husbandry and treatment of *Xenopus laevis* were approved by the Regierungspräsidium Freiburg, Germany. Mice were kept in accordance with the National Institutes of Health guidelines for the care and use of laboratory animals. All experiments on mice were approved by the Ethics Board of the Lower Saxony State Office for Consumer Protection and Food Safety. Murine embryos for gene expression analysis were derived from matings of wild-type mice with NMRI background. For timed pregnancies, vaginal plugs were checked in the morning after mating, and noon was defined as embryonic day (E) 0.5. Embryos were dissected in phosphate-buffered saline (PBS; Merck, Darmstadt, Germany) and fixed in 4% paraformaldehyde in PBS followed by dehydration using increasing methanol concentrations, i.e., incubation in 25%, 50%, and 75% methanol for 1 h each. Fixed embryos were stored in 100% methanol at −20 °C prior to in situ hybridization analyses.

### WES and targeted *GDF6* sequencing

WES was performed on whole-blood DNA of one patient–sibling–parents index family, 30 additional patients with renal malformations, and 74 control individuals using the SureSelectXT Human All Exon V4 target enrichment kit (Agilent, Santa Clara, CA, USA) on a HiSeq 2000 (Illumina, San Diego, CA, USA) sequencer or the SureSelectXT Human All Exon V5+UTRs target enrichment kit (Agilent) on a HiSeq 2500 (Illumina) sequencer. All samples were sequenced to a mean target coverage of >50×. Sequencing data were aligned to the human reference genome (hg19) using the Biomedical Genomics Workbench (Qiagen, Hilden, Germany). WES data of the index family were annotated and prioritized using Ingenuity Variant Analysis (Qiagen) and our in-house NGS data analysis workflow as described in “Results” and summarized in Supplementary Tables 2 and 3. Using conventional chain termination protocols and a 3130XL Genetic Analyzer (Thermo Fisher Scientific, Waltham, MA, USA), all coding exons and adjacent intronic regions of *GDF6* were analyzed for sequence variants in 163 further patients with kidney anomalies, selected *GDF6* variants identified by WES were verified, and familial segregation analysis was done (oligonucleotide sequences are given in Supplementary Table 4). Nucleotide numbering of the identified variants reflects the nucleotide position in the coding sequence of human *GDF6* mRNA (https://www.ncbi.nlm.nih.gov/nuccore/NM_001001557.4) (Supplementary Fig. 1).

### Immunohistochemistry, RNA in situ hybridization, CRISPR/Cas9 genome engineering and cellular assays, knockdown and rescue experiments in *Xenopus laevis*

Procedures are described in “Supplementary materials” (including Supplementary Tables 5–8).

### Statistical analysis

Statistical analysis was done using MATLAB and Statistics Toolbox Release 2018b (The MathWorks, Inc., Natick, MA, USA). Student’s *t*-test or Fisher’s exact test were used, as applicable, and *p* values are indicated (*p < 0.05, **p < 0.01, and ***p < 0.001).

## Results

### Using WES, a rare heterozygous *GDF6* missense variant, c.746C>A p.(Ala249Glu), was detected in the index patient

Under the assumption that NGS techniques are particularly successful in identifying the genetic cause in patients with syndromic CAKUT, we applied WES to whole-blood DNA of female patient F006.II.1 with a renal malformation (i.e., crossed fused renal ectopia) as well as skeletal (e.g., scoliosis, fused and butterfly vertebrae) (Fig. 1a–d), auricular (i.e., auricle dysplasia and aplasia of the external auditory canal), ocular (e.g., anisometropia), and other extrarenal anomalies, and of her mother, father, and sister who were not affected by renal anomalies. As no rare de novo, homozygous, or compound-heterozygous variants predicted to be disease causing could be detected in the high-quality exome data of patient F006.II.1, variants were prioritized using the strategy summarized in Supplementary Table 2. Prioritization of high-quality variants of patient F006.II.1 by seriousness, rareness, exclusiveness, and localization in genes (*n* = 207) reported to be mutated in at least one patient with syndromic CAKUT according to our in-house gene list yielded five rare (minor allele frequency (MAF) ≤ 1%) non-silent variants not present in controls and predicted to be disease causing by at least one prediction tool (MutationTaster, SIFT, or PolyPhen-2) (Supplementary Tables 2 and 3). One of these five variants, the *GDF6* variant c.746C>A p.(Ala249Glu), was presumed causative in patient F006.II.1 because it is reported to be disease causing by the HGMD Professional (v2018.2; Qiagen) and ClinVar (https://www.ncbi.nlm.nih.gov/clinvar/) databases in patients with skeletal and ocular anomalies [17–22] matching the patient’s extrarenal phenotype (Supplementary Table 9). By direct sequencing, the *GDF6* c.746C>A variant was confirmed to be heterozygous in the patient as well as in her sister and mother, both affected by the same mild auricular anomaly (Fig. 1h). The c.746C>A variant is located in the second exon of *GDF6* and occurs with a MAF of 0.001948 in the global population cohort and a MAF of 0.003783 in the non-Finnish European cohort of the Genome Aggregation Database (gnomAD v2.1.1) (Table 1). The amino acid alanine at position 249 is evolutionary conserved and located in the propeptide/prodomain of GDF6 (Supplementary Fig. 1). Based on the ACMG/AMP 2015 guidelines [23], we classified the c.746C>A variant as “pathogenic” (Table 1).

**Table 1:** Rare heterozygous variants in the *GDF6* gene predicted to be “deleterious” identified in 3 of 194 patients with renal anomalies.

Patient, gender, origin	Chromosomal position^a^, SNP number	Nucleotide change, amino acid change	MAF according to gnomAD^b^	Predictions			Inheritance	Renal phenotype	Extrarenal phenotype	ClinVar associations	Clinical interpretation and ACMG/AMP codes [23]
				MutationTaster	SIFT	PolyPhen-2					
N038.II.1, female, Norway (African mother from Ghana, Caucasian father from UK)	8:97172809, rs139075817	c.112G>C p.(Gly38Arg)	0.000656 0.000000 0.007016	“Polymorphism”	“Damaging”	“Possibly damaging”	Not paternal	Bilateral kidney hypodysplasia requiring kidney transplantation at age 5 years	Features of Klippel–Feil syndrome 1 (i.e., high-arched palate), macrocephaly, short narrow palpebral fissures	Not reported	“Uncertain significance” (PS3_strong; PP2_supporting; PP3_supporting; PP4_supporting)
F006.II.1, female, Germany	8:97157413, rs121909352	c.746C>A p.(Ala249Glu)	0.001948 0.003783 0.000295	“Disease causing”	“Tolerated”	“Benign”	Maternal	Left-sided crossed fused renal ectopia with hydronephrosis, megaureters and VUR	Features of Klippel–Feil syndrome 1 (i.e., vertebral segmentation defects including fusions, scoliosis, left-sided auricle dysplasia, and aplasia of the external auditory canal), anal atresia, rectovestibular fistula, small ventricular septal defects, patent *foramen ovale*, anisometropia, suspected microphthalmia	Klippel–Feil syndrome 1, isolated microphthalmia, Leber congenital amaurosis	“Pathogenic” (PS1_strong; PS3_strong)
H435.II.1, male, North Macedonia	8:97157413, rs121909352	c.746C>A p.(Ala249Glu)	0.001948 0.003783 0.000295	“Disease causing”	“Tolerated”	“Benign”	Paternal	Left-sided kidney hypodysplasia, nonfunctional on DMSA scan	Features of Klippel–Feil syndrome 1 (i.e., scoliosis, high-arched palate), lop ears, prognathism, short philtrum, short frenulum	Klippel–Feil syndrome 1, isolated microphthalmia, Leber congenital amaurosis	“Pathogenic” (PS1_strong; PS3_strong)

NCBI reference sequence: NM_001001557.4. Ensembl transcript ID: ENST00000287020.

Variants were submitted to ClinVar (accession numbers SCV001334266-SCV001334268; http://www.ncbi.nlm.nih.gov/clinvar/).

*SNP* single-nucleotide polymorphism, *MAF* minor allele frequency in global/non-Finnish European/African population according to gnomAD v2.1.1.

^a^According to GRCh37/hg19.

^b^Genome Aggregation Database v2.1.1.

### Heterozygous *GDF6* missense variants were detected in a total of three of 194 patients (1.6%) with renal malformations and three of 56 patients (5.4%) with renal plus skeletal, ocular or auricular anomalies

Having identified a *GDF6* variant previously associated with skeletal and ocular anomalies in a patient presenting with these phenotypes and additionally with a renal malformation, we explored the frequency of rare *GDF6* variants in a cohort of patients with renal anomalies. A total of 193 additional cases were either analyzed by WES (30 patients) or targeted sequencing of the *GDF6* gene (163 patients). By targeted sequencing, we identified the *GDF6* variant c.746C>A in an additional patient, H435.II.1 from North Macedonia, presenting with a hypodysplastic left kidney (Fig. 1f, i) that was nonfunctional on DMSA scan, mild skeletal, and auricular phenotypes, i.e., torsion scoliosis, high-arched palate, and lop ears (Table 1). The *GDF6* c.746C>A variant was detected in a heterozygous state in the patient, his father who presented with short stature, his brother who had a high-arched palate and lop ears, and a 1-year-old sister who was not further examined (Fig. 1i). Another rare heterozygous *GDF6* missense variant, c.112G>C p.(Gly38Arg), was detected by targeted sequencing in female patient N038.II.1 presenting with bilateral renal hypodysplasia (Fig. 1g) requiring kidney transplantation at age 5 years in addition to skeletal, i.e., macrocephaly and high-arched palate, and ocular, i.e., short narrow palpebral fissures, phenotypes (Fig. 1j and Table 1). As the c.112G>C variant was not inherited from her Caucasian father, it may be a de novo variant or have been inherited from her African mother (Fig. 1j) from whom no DNA sample was available for clarification. Located in the first exon of *GDF6* (Supplementary Fig. 1), the c.112G>C variant occurs with a MAF of 0.000656 in the global population cohort, a MAF of 0.007016 in the African population cohort, and is not present in the non-Finnish European cohort of the gnomAD database v2.1.1 (Table 1). While the c.112G>C variant was of “uncertain significance” based on the ACMG/AMP 2015 guidelines [23], the prediction tools SIFT and PolyPhen-2 rated it as “damaging” or “possibly damaging” (Table 1). The amino acid glycine at position 38 is evolutionary highly conserved. In total, we detected rare heterozygous *GDF6* missense variants predicted to be disease causing in 3 of 194 families with renal anomalies (1.6%). Clinical or radiological reevaluation of the skeleton, eye or ear revealed skeletal, ocular, or auricular abnormalities in 56 of the 194 patients with renal anomalies analyzed. As the three *GDF6* variant carriers were among these patients, 3 of 56 patients (5.4%) with renal plus skeletal, ocular, or auricular features carried *GDF6* variants with a disease-causing prediction, a significant frequency increase compared with that in renal anomaly patients without abnormalities of the skeleton, eye, or ear (0/138, *p* = 0.0231, Fisher’s exact test).

### *GDF6* is expressed in the infant human kidney, and in the developing murine urogenital system and pronephros of *Xenopus laevis*

**Fig. 2:**
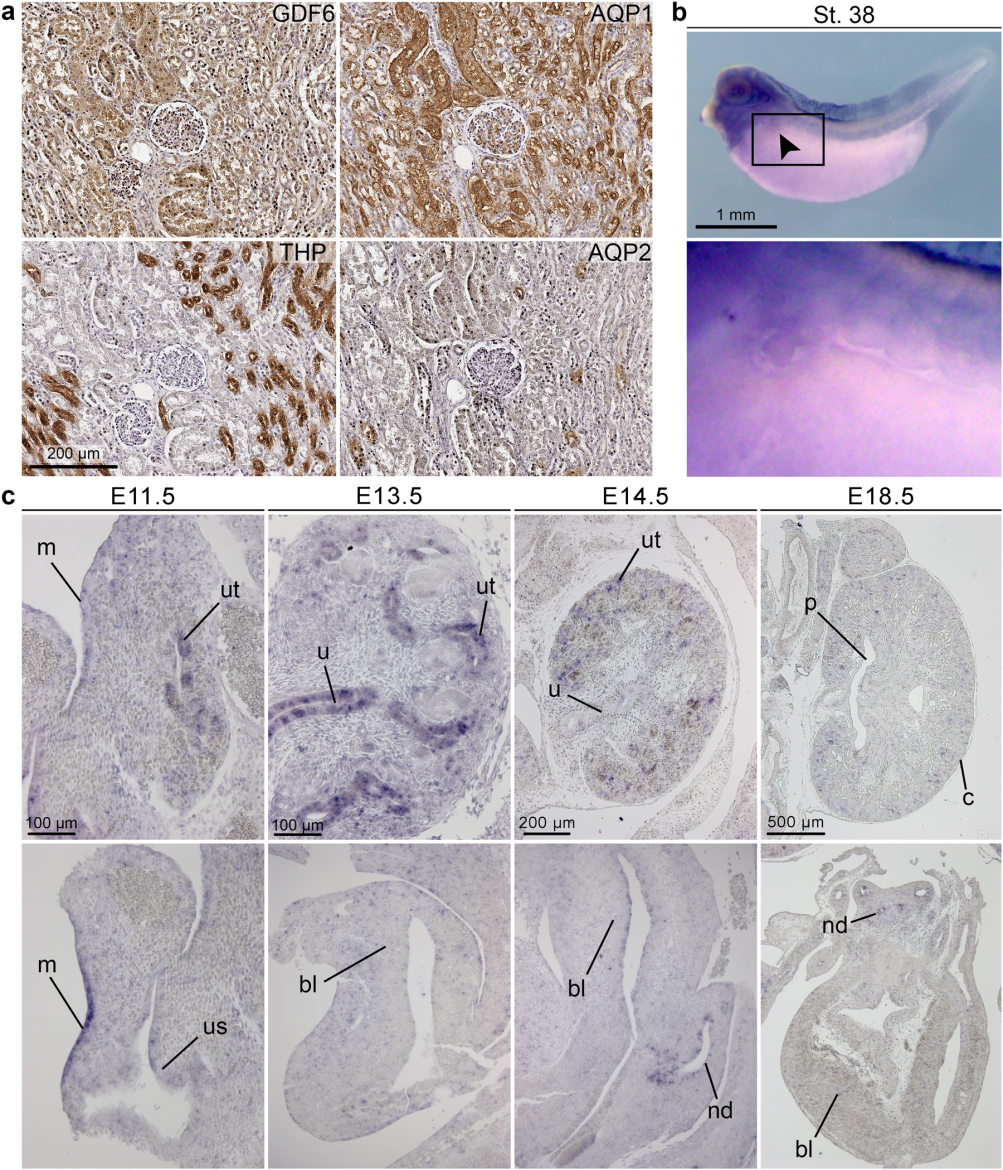
*GDF6* is expressed in the infant kidney, during pronephros development in *Xenopus laevis* and murine urinary tract development. **a** By immunohistochemical detection of GDF6 and the marker proteins aquaporin-1 (AQP1, straight and convoluted proximal tubule and thin descending limb of loop of Henle), aquaporin-2 (AQP2, collecting duct), and Tamm-Horsfall protein (THP, thick ascending limb of loop of Henle and distal tubule) in a normal human infant kidney section, GDF6 localized most prominently to proximal tubules. **b** By whole-mount RNA in situ hybridization in *Xenopus laevis* at stage 38, *gdf6* was expressed in the tissue surrounding the pronephric tubule (arrow, enlarged image) as well as in the developing eye, branchial arches, notochord, and neural tube. **c** RNA in situ hybridization analysis on sagittal sections of the murine kidney and bladder from E11.5 to E18.5. *Gdf6* expression was present in all compartments of the developing ureteric tree, including the ureteric tips (ut) and the ureter (u), at E11.5 to E14.5, dropped after onset of collecting duct differentiation, and was barely detectable at E18.5 (p pelvis, c cortex). In the lower urogenital tract, *Gdf6* expression was found in the mesothelial lining (m) of the abdominal cavity at E11.5, in the undifferentiated urothelium of the urogenital sinus (us) and the bladder (bl) until E14.5, and in the nephric duct (nd) of male embryos at all analyzed stages (data partly not shown). Sections from three independent murine specimens were analyzed.

Having detected rare *GDF6* variants in patients with renal anomalies, we went on to explore a possible role for *GDF6* in kidney development by determining the expression pattern of *GDF6* in the infant human kidney as well as in *Mus musculus* and *Xenopus laevis* during development. GDF6 protein expression was detected in the human infant kidney, most prominently in proximal tubules, by immunohistochemistry (Fig. 2a). During lower vertebrate development, expression of *gdf6* was observed in the area of the pronephros, the embryonic kidney of *Xenopus laevis*, with particular enhancement in the tissue just adjacent to the pronephric tubule at stage 38 by whole-mount RNA in situ hybridization. Expression at this stage was also detected around the eye vesicle, branchial arches, notochord, and neural tube of *Xenopus laevis* embryos (Fig. 2b). During murine urogenital system development, *Gdf6* mRNA was present in the ureteric tips at E11.5 shortly after onset of metanephros development. At E13.5, expression of *Gdf6* in the kidney reached its peak and was found in all compartments, i.e., the ureter and ureteric tips, of the developing ureteric tree. After onset of collecting duct differentiation at E14.5, *Gdf6* transcript levels decreased in the kidney, and renal expression was barely detectable at E18.5. At E11.5, *Gdf6* was also detectable in the mesothelial lining of the abdominal cavity and weakly in the epithelium of the urogenital sinus, which develops into the bladder. Expression in the bladder urothelium vanished after E14.5. *Gdf6* mRNA was also found in the nephric duct or differentiated *vas deferens* of male embryos at all analyzed stages (Fig. 2c, data partly not shown).

### Knockout of *Gdf6* in murine inner medullary collecting duct (mIMCD3) cells impairs cell migration, an effect rescued by expression of wild-type not mutant *GDF6* in *Gdf6*^−/−^ mIMCD3 cells

Next, we generated an in vitro test system to determine whether *GDF6* impacts cell migration, a central process in development, and to assess whether the identified *GDF6* variants affect this function. Using CRISPR/Cas9 technology, a single guide RNA targeting the first coding exon of *Gdf6* was applied to knockout *Gdf6* in mIMCD3 cells. Two *Gdf6*^*−/−*^ mIMCD3 cell clones with frameshift variants predicted to result in premature stop codons and nonfunctional proteins were identified, i.e., clone 32 harboring the homozygous *Gdf6* variant c.377_378delCA p.(Ser126Cysfs*2), and clone 34 containing the biallelic *Gdf6* variants c.373_376delAAGT p.(Lys125Glnfs*9) and c.377_378delCA p.(Ser126Cysfs*2) (Supplementary Fig. 2). A mIMCD3 cell clone with no mutational event in *Gdf6* (clone 2, *Gdf6*^*+/+*^) was also identified and used as a control (Supplementary Fig. 2). No differences in cell viability were observed when comparing mIMCD3 cells and *Gdf6*^*+/+*^ mIMCD3 cell clone 2 with the *Gdf6*^*−/−*^ mIMCD3 cell clones 32 or 34 using a cell viability assay (Supplementary Fig. 3).

**Fig. 3:**
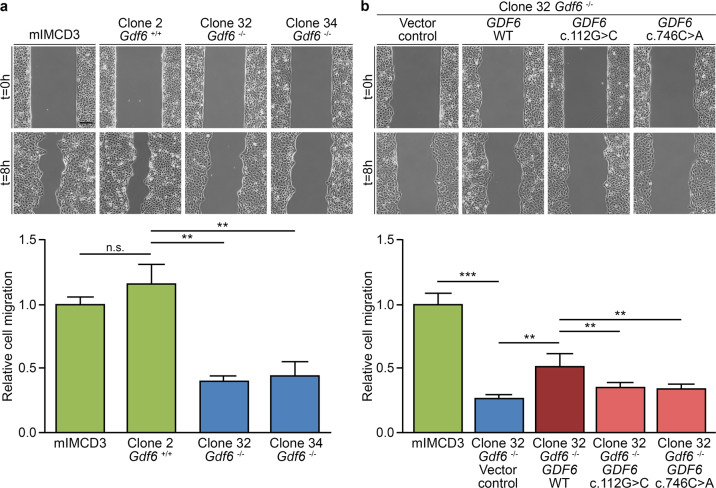
Knockout of *Gdf6* impacts migration of murine IMCD3 cells, an effect partially reversed by expression of wild-type not mutant *GDF6*. **a** Relative to mIMCD3 cells, migration of *Gdf6*^*−/−*^ mIMCD3 cells (clones 32 and 34) was significantly decreased compared with *Gdf6*^*+/+*^ mIMCD3 cell clone 2. **b** The effect of *Gdf6* knockout in mIMCD3 cell clone 32 expressing empty vector (vector control) relative to mIMCD3 cells was partially rescued by stable expression of human wild-type (WT) *GDF6* in mIMCD3 cell clone 32. Conversely, relative cell migration of *Gdf6*^*−/−*^ mIMCD3 cell clone 32 stably expressing *GDF6* c.112G>C or c.746C>A variants was significantly reduced compared with *Gdf6*^*−/−*^ mIMCD3 cell clone 32 stably expressing wild-type *GDF6*. All results are mean ± SD from three independent experiments. Scale bar: 150 µm. N.s. not significant; **p < 0.01; ***p < 0.001.

A time series analyzing mIMCD3 cell migration in a wound healing assay resulted in a reduction of the cell-free area by 50% after 8 h (Supplementary Fig. 4). Therefore, in subsequent analyses the cell-free areas were determined and compared at 0 h and 8 h. While migration of mIMCD3 cells and *Gdf6*^*+/+*^ mIMCD3 cell clone 2 did not differ significantly, migration of *Gdf6*^*−/−*^ mIMCD3 cell clones 32 and 34 was significantly decreased compared with *Gdf6*^*+/+*^ mIMCD3 cell clone 2 (*p* = 0.002 and *p* = 0.005, respectively; Fig. 3a), thereby providing evidence that *Gdf6* knockout impacts cell migration. Migration was also significantly impaired in *Gdf6*^*+/−*^ mIMCD3 cell clone 30 versus *Gdf6*^*+/+*^ mIMCD3 cell clone 2 (*p* = 0.041; Supplementary Fig. 5), indicating an effect on migration of heterozygously mutated cells, a cellular model for patients with heterozygous *GDF6* variants. A significant increase in migration of *Gdf6*^*−/−*^ mIMCD3 cell clone 32 stably transfected with a wild-type *GDF6* expression construct was detected compared with *Gdf6*^*−/−*^ mIMCD3 cell clone 32 transfected with empty vector (*p* = 0.007; Fig. 3b and Supplementary Fig. 6), showing that re-expression of human wild-type *GDF6* can rescue the reduced migration. In contrast, migration of *Gdf6*^*−/−*^ mIMCD3 cell clone 32 transfected with constructs expressing *GDF6* variants c.112G>C or c.746C>A versus *GDF6* wild-type was significantly reduced (*p* = 0.003 and *p* = 0.008, respectively; Fig. 3b and Supplementary Fig. 6), demonstrating that the identified *GDF6* variants are not functional in this assay.

### Morpholino (MO) knockdown of *gdf6* in *Xenopus laevis* impairs pronephros development

**Fig. 4:**
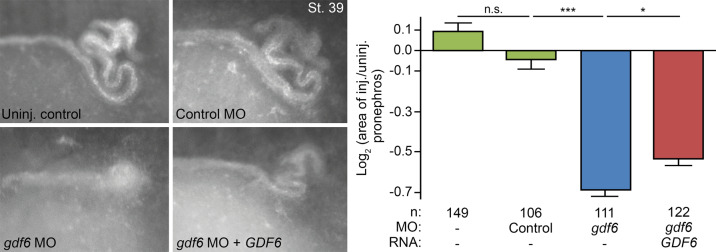
Morpholino (MO) knockdown of *gdf6* in *Xenopus laevis* impairs pronephros development. *Xenopus laevis* stage (st.) 39 tadpoles were unilaterally injected with a control MO or a translation-blocking *gdf6* MO with or without co-injection of *GDF6* mRNA, and stained with fluorescein-labeled lectin to visualize the pronephros. The ratios of the pronephros areas of the injected (inj.) and the uninjected (uninj.) side of the embryo were calculated and log_2_ transformed. Knockdown with the *gdf6* MO led to a significantly reduced pronephros area. This effect was significantly rescued by the co-injection of *GDF6* mRNA. Results are mean ± SEM from *n* embryos analyzed in four independent experiments. N.s. not significant; *p < 0.05; ***p < 0.001.

To explore a possible role of *gdf6* in renal development in vivo, we performed *gdf6* knockdown unilaterally using a specific *gdf6* antisense MO in *Xenopus laevis* tadpoles, and analyzed the developing pronephros, constituting the embryonic kidney in lower vertebrates, at stage 39 (Fig. 4). The pronephric area, calculated as log_2_ ratio of the injected and uninjected side of the tadpole, was significantly reduced after *gdf6* MO injection compared with control MO injection (*p* = 7.6 × 10^−13^; Fig. 4). This effect was partially rescued by co-injection of *GDF6* mRNA with *gdf6* MO that increased the pronephros area significantly compared with injecting *gdf6* MO alone (*p* = 0.035; Fig. 4). Thereby, we demonstrate a role for *gdf6* in pronephric tubule development in *Xenopus laevis*.

## Discussion

In the present study systematically investigating a role of *GDF6* in renal anomalies, we identified rare heterozygous *GDF6* variants in 1.6% of patients with kidney malformations. Initially, we found *GDF6* to be associated with renal malformation by an unbiased screen for germline variation in a patient with renal as well as skeletal, auricular, ocular, and other anomalies. This patient had been chosen for WES analysis because NGS technologies have been particularly successful in identifying causative genes in syndromic CAKUT [9]. Subsequently, we detected two further renal anomaly patients with rare heterozygous *GDF6* variants among the 193 patients additionally analyzed. In line with the extrarenal manifestations of our three renal anomaly patients carrying *GDF6* variants, variants in *GDF6* have previously been reported in patients with skeletal phenotypes, i.e., (i) Klippel–Feil syndrome (KFS) with vertebral segmentation defects frequently associated with scoliosis, rib abnormalities, and Sprengel’s deformity now known as KFS1 [17, 18], (ii) Chiari malformations [17, 24], and (iii) multiple synostoses syndrome including carpal and tarsal fusions [25–27], as well as ocular phenotypes, i.e., (i) microphthalmia [20–22], (ii) coloboma [18, 28], (iii) Leber congenital amaurosis or juvenile retinitis pigmentosa [19], and (iv) glaucoma [29]. By adding three patients with renal anomalies to the two patients previously reported [17, 18], a total of 5 of 86 (5.8%) individuals carrying rare *GDF6* variants described here and in the literature (Supplementary Table 9) are known to be affected by congenital kidney malformations. This finding suggests that renal sonography is warranted in patients carrying rare *GDF6* variants irrespective of the other abnormalities or disorders they may present with. Vice versa, *GDF6* mutational analysis may be advisable in patients with renal anomalies, in particular in those cases additionally affected by the skeletal and ocular phenotypes previously associated with rare *GDF6* variants and also present in the renal anomaly patients with *GDF6* variants of this study. Accordingly, the percentage of rare *GDF6* variants in patients with renal plus bone, eye, or ear abnormalities was 5.4%, significantly higher than in renal anomaly patients without these extrarenal manifestations. In this context, it is notable that we detected one *GDF6* variant, the missense variant c.746C>A, recurrently in renal anomaly patients, resulting in a significant frequency increase compared with the cohort of the 1000 Genomes Project. Previously, the *GDF6* c.746C>A variant was reported in patients with KFS-like skeletal anomalies [17, 18, 20], Chiari malformation [24], micro- or anophthalmia [20–22], and Leber congenital amaurosis or juvenile retinitis pigmentosa [19] (Supplementary Table 9), demonstrating that it can be associated with a spectrum of different phenotypes. In line with these findings, we detected skeletal anomalies in four of seven *GDF6* c.746C>A variant carriers from two families, and ocular anomalies in one carrier. Similar to kidney malformations that were present in two of the seven *GDF6* c.746C>A variant carriers here, penetrance is reduced for these abnormalities. Reduced penetrance is a known feature associated with renal anomalies. This is exemplified by a family with Stickler syndrome and a heterozygous nonsense variant in the *BMP4* gene encoding bone morphogenetic protein 4, a ligand related to GDF6, with renal dysplasia in only one of five family members carrying the *BMP4* variant [30].

*GDF6* encodes growth differentiation factor 6, a member of the BMP family within the transforming growth factor beta (TGF-β) superfamily of ligands that utilize type I and type II transmembrane serine–threonine kinase receptors [31]. Therefore, in *GDF6* variant carriers reduced penetrance for renal malformations may be explained by the complexity of TGF-β signaling comprising numerous ligands (around 30), receptors, and downstream interacting proteins [31], suggesting some redundancy. The GDF6 amino acid sequence consists of three domains. These are a signal peptide, a propeptide/prodomain, and a mature receptor-binding carboxy-terminal TGF-domain that is highly conserved between species, i.e., it is more than 90% identical in *Xenopus laevis* and mouse [32]. Interestingly, 74% of the *GDF6* variants detected in patients with different phenotypes (Supplementary Table 9), including the recurrent c.746C>A variant and the c.112G>C variant identified in renal anomaly patients here, affect amino acids located in the prodomain of the GDF6 sequence. Although it is less conserved, the prodomain of TGF-β superfamily ligands is known to regulate the synthesis, extracellular localization, and activity of these proteins [33]. The prodomain of BMP4, another member of the BMP family of ligands, for instance, is necessary to generate stable BMP4/7 heterodimers with enhanced bioactivity in vivo [34]. Therefore, it is not unexpected that although the c.746C>A variant affects an amino acid located in the prodomain of GDF6, the activity of variant GDF6 was significantly decreased in a *SOX9*-reporter luciferase assay [18], and amounts of variant pre-pro-protein and mature ligand were reduced in the media of transfected cells [19] compared with wild-type. Furthermore, we show here that impaired cell migration of murine *Gdf6* knockout cells from the renal inner medullary collecting duct is rescued by expression of wild-type *GDF6* but not of c.746C>A and c.112G>C variants, indicating that both are hypomorphic variants with respect to cell movements, a fundamental process in development.

BMP signaling is highly implicated in embryogenesis including nephrogenesis and development [35–38]. CAKUT phenotypes were observed with high penetrance in mice carrying a heterozygous or homozygous knockout for genes encoding BMP ligands such as BMP4 [39], BMP7 [40], and GDF11 [41]. Pleiotropic defects have been described as a result of *Gdf6* knockout, knockdown, or variation in mice, zebrafish, or *Xenopus laevis* including defects in joint, ligament, and cartilage formation causing carpal and tarsal fusions and coronal craniosynostosis [42], altered tail tendon fascicles [43], shorter lengths of digits and dermal flat bones in the skull [44], microphthalmia, anophthalmia, and coloboma [20, 45]. Whether urogenital tract anomalies exist has not previously been examined in these animal models. Based on the results from our expression and functional studies and the finding that other BMP ligands, such as BMP4, regulate the budding site and elongation of the developing mouse ureter [39], we propose that the rare *GDF6* variants detected in five renal anomaly patients here and previously [17, 18] may be causally related to their kidney malformation. This proposal is in line with the spectrum of renal abnormalities seen in *GDF6* variant carriers that includes renal agenesis [17] and renal hypodysplasia (two cases here), two anomalies also found in patients carrying *BMP4* variants [46]. Kidney agenesis or hypoplasia are potentially caused by defects in ureteric budding and branching of the ureteric tree induced by abberant BMP signaling as detected in *Bmp4* heterozygous knockout mice [39]. It is quite conceivable that such budding and branching defects giving rise to missing or small dysplastic kidneys also occur in human carriers of rare *GDF6* variants because the developing ureteric tree expresses *Gdf6*, and migration of collecting duct cells that originate from the ureteric tree is impaired by *Gdf6* knockout, as shown here. Similarly, pronephros size was reduced by *gdf6* knockdown in *Xenopus laevis*. We and others additionally observed kidney abnormalities involving renal fusions, i.e., a horseshoe kidney [18] and a crossed fused renal ectopia (one case here), in patients with rare *GDF6* variants, similar to the skeletal fusions seen (Supplementary Table 9). These data imply that BMP signaling is also linked to skeletal and renal fusions, similar to observations in a conditional *Bmp4* knockout mouse in which reduced BMP signaling resulted in hindlimb fusion [47]. According to an established view, renal fusions result as a consequence of abnormal renal ascent during embryogenesis [48], possibly connecting GDF6 to the migration of the kidneys as also suggested by our cell migration studies.

In summary, we identified rare heterozygous *GDF6* variants in 1.6% of all patients of our renal anomaly cohort, and in 5.4% of those patients additionally manifesting skeletal, ocular, or auricular abnormalities suggesting that *GDF6* is associated with human kidney malformations. The phenotype spectrum identified in renal anomaly patients with *GDF6* variants ranged from hypodysplasia to fusion. Furthermore, *Gdf6* expression in the murine developing ureteric tree, diminished migration of murine *Gdf6*^*−/−*^ collecting duct cells and impaired pronephros development after *gdf6* knockdown in *Xenopus laevis* may support a role of GDF6 in kidney development.

## Supplementary information

Supplementary material

Supplementary Table 3
