# Supplementary material for: Rare heterozygous GDF6 variants in patients with renal anomalies
Source: Eur J Hum Genet. 2020 Jul 31;28(12):1681–93. doi: 10.1038/s41431-020-0678-9 (PMC7784874; doi:10.1038/s41431-020-0678-9)
Supplement: Supplementary file 1 — Supplementary material [file 41431_2020_678_MOESM1_ESM.pdf]

Supplementary material

Rare heterozygous *GDF6* variants in patients with renal anomalies

Helge Martens,[1] Imke Hennies,[2] Maike Getwan,[3,4] Anne Christians,[1] Anna-Carina Weiss,[5] Frank Brand,[1] Ann Christin Gjerstad,[6] Arne Christians,[7] Zoran Gucev,[8] Robert Geffers,[9] Tomáš Seeman,[10] Andreas Kispert,[5] Velibor Tasic,[8] Anna Bjerre,[6] Soeren S. Lienkamp,[3,4] Dieter Haffner,[2] Ruthild G. Weber[1]

[1]Department of Human Genetics, Hannover Medical School, 30625 Hannover, Germany;
[2]Department of Pediatric Kidney, Liver and Metabolic Diseases, Hannover Medical School, 30625 Hannover, Germany;
[3]Department of Medicine, Renal Division, Medical Center, University of Freiburg, 79110 Freiburg, Germany;
[4]Institute of Anatomy, University of Zurich, 8057 Zurich, Switzerland;
[5]Institute of Molecular Biology, Hannover Medical School, 30625 Hannover, Germany;
[6]Division of Paediatric and Adolescent Medicine, Oslo University Hospital, 0424 Oslo, Norway;
[7]Institute of Pathology, Division of Neuropathology, Hannover Medical School, 30625 Hannover, Germany;
[8]Medical Faculty Skopje, University Children's Hospital, Skopje 1000, North Macedonia;
[9]Genome Analytics Research Group, Helmholtz Centre for Infection Research, 38124 Braunschweig, Germany;
[10]Department of Paediatrics, Second Faculty of Medicine, Charles University Prague, Praha 5, 150 06, Czech Republic

Correspondence: Ruthild G. Weber, Department of Human Genetics OE 6300, Hannover Medical School, Carl-Neuberg-Str. 1, D-30625 Hannover, Germany, Phone: +49 511 5327751, Fax: +49 511 53218520, E-mail: weber.ruthild@mh-hannover.de

These authors jointly supervised this work: Dieter Haffner, Ruthild G. Weber

Supplementary materials and methods

Immunohistochemistry on human normal kidney sections

Immunohistochemistry was performed on sections of formalin-fixed paraffin-embedded normal human kidney from a male infant removed at autopsy. Sections were mounted on SuperFrost slides (Langenbrinck, Emmendingen, Germany). The following primary antibodies and dilutions were used: GDF6 rabbit polyclonal antibody (1:20, #PA5-14394; Thermo Fisher Scientific, Waltham, MA, USA), THP rabbit polyclonal antibody (1:250, #sc-20631; Santa Cruz Biotechnology, Inc., Santa Cruz, CA, USA), AQP1 rabbit polyclonal antibody (1:4000, #AB2219; Merck, Darmstadt, Germany), and AQP2 rabbit polyclonal antibody (1:100, #sc-28629; Santa Cruz Biotechnology). Subsequently, slides were treated with a horseradish peroxidase-labeled F(ab')2-goat anti-rabbit secondary antibody (1:200, #A24537; Thermo Fisher Scientific) followed by incubation with Liquid DAB+ Substrate (Dako North America, Carpinteria, CA, USA) and counterstaining with Mayer’s hemalum solution. Slides were scanned using the Aperio AT2 Scanner (Leica Microsystems, Wetzlar, Germany), and digital images were processed using ImageScope v11 software (Leica Microsystems).

RNA *in situ* hybridization analysis in *Xenopus laevis* and murine development

Gene expression analysis of *gdf6* in *Xenopus laevis* was done using whole-mount RNA *in situ* hybridization as described previously (Sive et al. 2000). A *Xenopus laevis gdf6* construct for whole-mount *in situ* hybridization published earlier (Schille et al. 2016) was used in this experiment. The construct was linearized with *Spe*I and transcription performed with T7 RNA polymerase (Roche, Basel, Switzerland) for antisense probe synthesis. Hybridized probe was detected with an alkaline phosphatase-conjugated secondary antibody (Roche). To determine the expression of *Gdf6* in the development of the murine urogenital system, RNA *in situ* hybridization analysis on sections of the kidney and the bladder of NMRI wildtype embryos from E11.5 to E18.5 was carried out following a standard protocol (Moorman et al. 2001). For each stage, at least three specimens were analyzed. Stained sections were

documented using a Leica DM5000 microscope and a Leica DFC350FX digital camera (Leica Microsystems) and processed with Adobe Creative Cloud.

Cell culture and transient transfection

Murine inner medullary collecting duct (mIMCD3) cells were cultured in DMEM/Ham's F-12 (1:1) medium (Merck) supplemented with 10% fetal bovine serum, 100 units/ml penicillin, and 100 µg/ml streptomycin (Thermo Fisher Scientific). Cell cultures were maintained at 37°C in an incubator with a humidified atmosphere of 5% CO_2 and 95% air. Transient transfection was done using Lipofectamine 3000 transfection reagent (Thermo Fisher Scientific) following standard procedures.

CRISPR/Cas9 genome engineering of mIMCD3 cells

A protocol by Ran et al. using the CRISPR/Cas9-mediated system for RNA-programmable genome editing (Ran et al. 2013) was modified to generate a *Gdf6*-knockout cellular model using mIMCD3 cells. Using a bioinformatic tool (http://www.crispor.tefor.net), the single guide RNA (sgRNA) target sequence 5’-AGT GAT CGT ATT AGC TGA CT-3’ targeting exon 1 of *Gdf6* was selected, and sense and antisense oligonucleotides were synthesized (Eurofins Genomics, Ebersberg, Germany) containing a 5'-CAC CG-3' cloning overhang at the 5'-end and a 5'-TGG-3' protospacer adjacent motif (PAM) site at the 3'-end. This sgRNA target sequence is predicted to have no exonic off-target binding regions. Complementary oligonucleotides were cloned into the pSpCas9(BB)-2A-GFP plasmid (Addgene plasmid #48138) containing the sgRNA scaffold and expression cassettes for Cas9 and GFP using a directional topoisomerase cloning protocol. Murine IMCD3 cells were transiently transfected with the resulting construct. GFP-positive cells were isolated 24 hours after transfection at the Cell Sorting Core Facility of Hannover Medical School using a MoFlo XDP cell sorter (Beckman-Coulter, Brea, MA, USA). DNA was extracted from 36 single cell clones after cell expansion using the innuPREP DNA Mini Kit (Analytik Jena, Jena, Germany), and sequence analysis of *Gdf6* exon 1 was done using oligonucleotides listed in Supplementary Table 4.

For allele-specific sequence analysis of clone 34, PCR amplicons of genomic DNA containing the *Gdf6* exon 1 target locus were cloned into the pcDNA3.1 vector (Invitrogen, Carlsbad, CA, USA) using restriction enzyme-based cloning. After transformation, 20 *Escherichia coli* colonies were amplified, plasmid DNA was isolated using the NucleoSpin Plasmid Mini Kit (Macherey-Nagel, Düren, Germany), and *Gdf6* exon 1 inserts were sequenced (Supplementary Fig. 2). Consequences of nucleotide alterations were predicted using SnapGene Viewer (version 4.2.5; GSL Biotech, Chicago, IL, USA).

Cloning of *GDF6* expression constructs and stable transfection of mIMCD3 cells

The human full-length *GDF6* coding sequence was amplified by PCR from cDNA synthesized from total RNA of an individual without *GDF6* variants using the SuperScript III First-Strand Synthesis Kit (Thermo Fisher Scientific). pUNO1-*GDF6* expression constructs were generated by cloning of human *GDF6* cDNA into the pUNO1-mcs vector (InvivoGen, San Diego, CA, USA) using the In-Fusion HD Cloning Kit (Takara Bio, Kusatsu, Japan) and the oligonucleotides listed in Supplementary Table 5. The *GDF6* expression construct was used as a template to insert (i) the c.112G>C variant identified in patient N038.II.1 and (ii) the c.746C>A variant identified in patient F006.II.1 and H435.II.1 using the oligonucleotides listed in Supplementary Table 6. The Phusion Site-Directed Mutagenesis Kit (Thermo Fisher Scientific) was used for mutagenesis. The inserts of the generated constructs were verified using conventional chain termination protocols.

To analyze *GDF6* variant-specific effects, mIMCD3 cell clone 32 (*Gdf6*$^{-/-}$) was transfected with pUNO1 expression constructs containing no insert (vector control), human *GDF6* wildtype or mutant *GDF6* harboring the c.112G>C or c.746C>A variants. Selection of cells containing the pUNO1 expression constructs was done by adding 5 µg/µl blasticidin S (Thermo Fisher Scientific) to the cell culture medium starting 48 h after transfection for at least 14 days. Subsequently, genomic DNA was extracted from mIMCD3 cells using the innuPREP DNA Mini Kit (Analytik Jena). Generation of plasmid-specific amplicons by PCR using oligonucleotides listed in Supplementary Table 7 as detected by agarose gel

electrophoresis demonstrated stable transfection of mIMCD3 cell clone 32 (*Gdf6*$^{-/-}$) with the pUNO1 expression constructs (Supplementary Fig. 6).

Cell viability assay

Viability of mIMCD3 cells was measured using the CellTiter 96 AQueous One Solution Cell Proliferation Assay (MTS assay; Promega, Madison, WI, USA). Cells were seeded in 96-well plates at 1.2 x 10^4 cells per well and cultured for 24 h. After adding 20 µl of MTS solution and incubating at 37°C for 2 h, light absorbance at 490 nm was detected using the FLUOstar Omega Plate Reader (BMG Labtech, Ortenberg, Germany). Three independent experiments were performed per cell line and mean values were calculated.

Cell migration assay

Cell migration capacity was analyzed using a wound healing assay. Murine IMCD3 cells were cultured in 35 mm µ-Dishes with 3-well culture inserts (Ibidi, Martinsried, Germany). In each well, 3.0x10^4 cells were seeded and cultured for 24 h to reach full confluency. After removal of the culture insert, cells were gently washed with PBS (Merck) and treated with 30 µg/µl mitomycin C (Santa Cruz Biotechnology) in standard culture medium for the first 2 h to inhibit cell proliferation. Four images per well were documented initially and 8 h after removal of the culture inserts using a DM IL LED FLUO microscope (Leica Microsystems) equipped with an EC3 camera (Leica Microsystems). For each image, the area of the cell free gap was measured using Fiji/ImageJ (Schindelin et al. 2012; Schneider et al. 2012) and mean values per well were calculated. Cell migration was determined as the difference between mean gap area at time points 0 and 8 h. For each cell line, mean values were calculated from three independent experiments, and cell migration relative to mIMCD3 cells was determined.

Knockdown and functional rescue experiments in *Xenopus laevis*

A translation-blocking morpholino oligonucleotide (MO) for *Xenopus laevis gdf6* (5'-GGC TCC TGT ATG TAT CCA TTA GCG G-3') was designed by and ordered from Gene Tools

(Philomath, Oregon, USA). Full-length human *GDF6* was cloned into a VF10 vector using *Mlu*I and *Not*I cloning sites and linearized for *GDF6* mRNA synthesis using *Sal*I. The T7 RNA polymerase (Roche) was used for *in vitro* transcription with the mMESSAGE mMACHINE Kit (Ambion, Kassel, Germany). A random control MO (5'-CCT CTT ACC TCA GTT ACA ATT TAT A-3') from Gene Tools was used as control for the *gdf6* MO and *memRFP* mRNA (plasmid kindly provided by J. Wallingford, Austin, Texas, USA) as control for *GDF6* mRNA.

Xenopus laevis embryos were transferred to 2% Ficoll dissolved in 0.3x Marc's Modified Ringer's solution (MMR) for injections (Sive et al. 2000). Subsequently, 13.6 ng *gdf6* MO in 10 nl per injection were injected into the ventrolateral region of 4-8 cell stage embryos targeting the later pronephros. For the rescue experiment, *gdf6* MO was co-injected with 0.4 ng *GDF6* mRNA that lacks the binding site of the *gdf6* MO. To be able to sort embryos for correct injections in later stages, 2 ng fluorescein-conjugated dextran (70,000 MW; Invitrogen) was co-injected in all experiments. Embryos were kept in 0.3x MMR buffer with gentamycin (0.05 mg/ml) until the desired stage was reached followed by fixation in MEMFA (0.1 mol/L MOPS, 2 mmol/L EGTA, 1 mmol/L $MgSO_4$, 3.7% formaldehyde, pH 7.4) for 1.5 h at room temperature and bleaching with H_2O_2 and methanol (1:2) for 1-2 h. The pronephros was visualized using immunofluorescence with fluorescein-labeled *Lycopersicon esculentum* lectin (1:100 dilution; Vector Laboratories, Burlingame, CA, USA). Imaging was done using SteREO Discovery.V8 and ZEN 2011 (blue edition) microscopes (Zeiss, Oberkochen, Germany). Pronephros area (bounding box of ventral-dorsal and anterior-posterior distance) was measured using ImageJ, and the ratio of injected versus uninjected side was calculated.

Supplementary figures

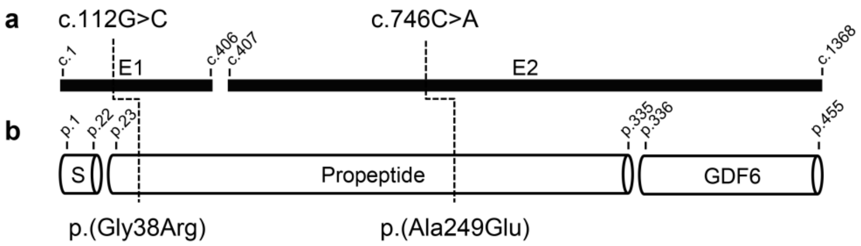

Supplementary Fig. 1 Location of two *GDF6* variants identified in three patients with renal anomalies in this study. **a** Schematic view of coding sequence of human *GDF6* mRNA (NM_001001557.4). E1, exon 1; E2, exon 2. **b** The human GDF6 protein sequence consists of a N-terminal signal peptide sequence (S), a propeptide/prodomain sequence, and a C-terminal mature protein sequence (GDF6) (https://www.uniprot.org/uniprot/Q6KF10). Both variants detected in this study are located in the GDF6 propeptide. Structures were designed using ExPASy prosite MyDomains (https://prosite.expasy.org/mydomains).

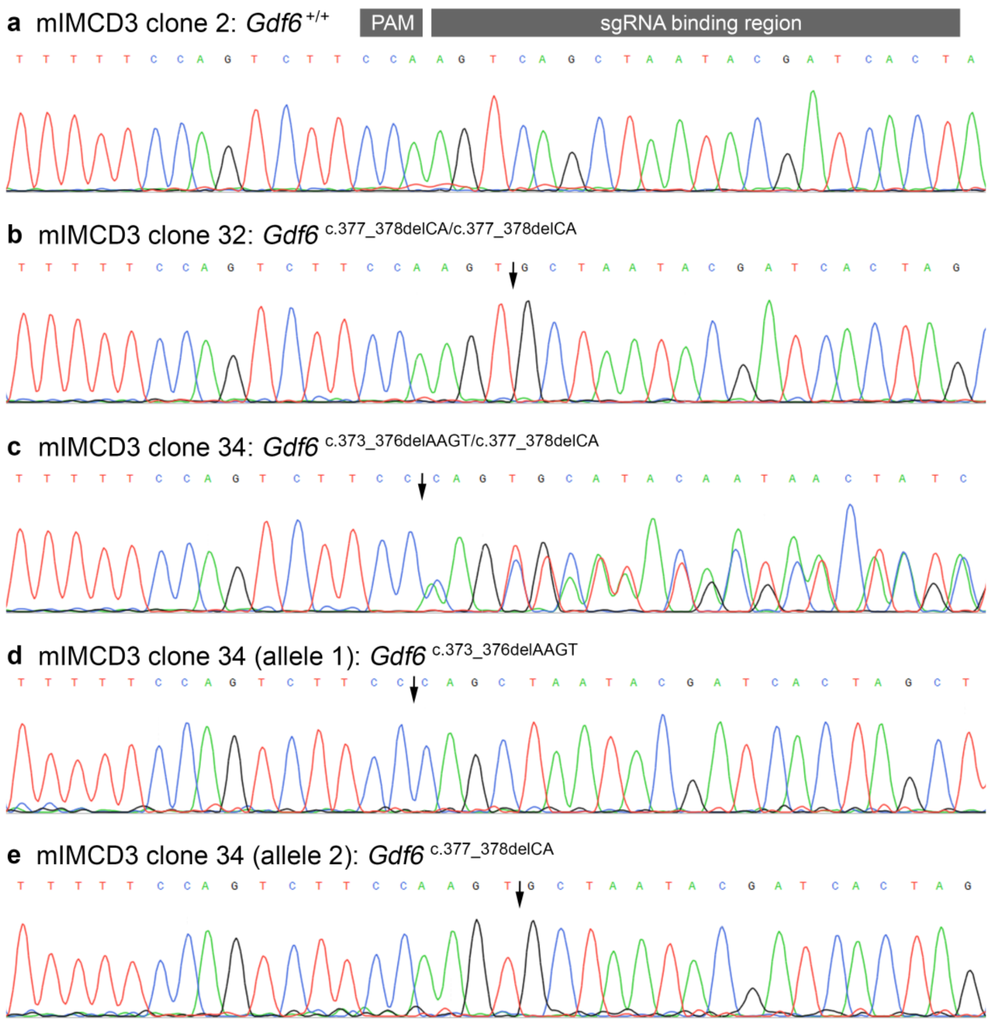

Supplementary Fig. 2 Electropherograms of the murine *Gdf6* sequence in exon 1 targeted by the single guide RNA (sgRNA) used for CRISPR-Cas9 genome engineering in murine IMCD3 cell lines. **a** Clone 2 revealed no variation within the murine *Gdf6* sequence and was used as a control. **b** In clone 32, the homozygous deletion c.377_378delCA causing a frameshift, p.(Ser126Cysfs*2), predicted to result in a premature stop codon and a non-functional protein was detected, thus identifying knockout of *Gdf6*. **c** The *Gdf6* sequence variation in clone 34 was biallelic. **d-e** *Gdf6* allele separation in clone 34 revealed the deletion c.373_376delAAGT on one allele and the deletion c.377_378delCA on the other allele both causing frameshifts predicted to lead to non-functional proteins, p.(Lys125Glnfs*9) and p.(Ser126Cysfs*2), or nonsense-mediated mRNA decay, thereby demonstrating knockout of *Gdf6* in clone 34. The protospacer adjacent motif (PAM) for Cas9 binding and the sgRNA target region are shown in **a**.

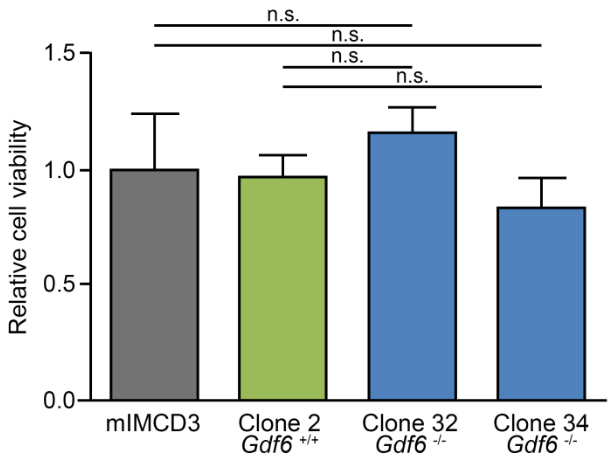

Supplementary Fig. 3 Cell viability measured by MTS assay 24 h after seeding was not significantly different when comparing mIMCD3 cells and *Gdf6*$^{+/+}$ mIMCD3 cell clone 2 with *Gdf6*$^{-/-}$ mIMCD3 cell clones 32 or 34; results are mean ± SD from three independent experiments.

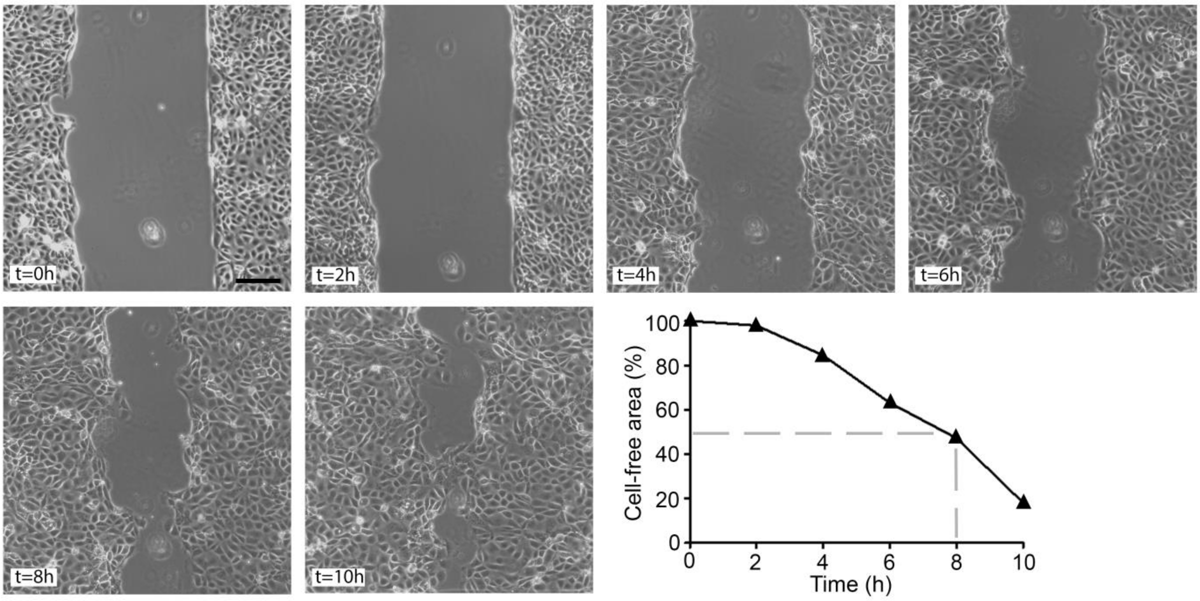

Supplementary Fig. 4 Time series of mIMCD3 cell migration. The speed of mIMCD3 cell migration and, thereby, the optimal duration of a wound healing assay using these cells was determined at intervals of 2 h for a total time period of 10h. After 8 h, the gap size was reduced by 50%. Therefore, this time point was chosen for subsequent analyses (see Fig. 3 and Supplementary Fig. 5). Scale bar: 150 µm.

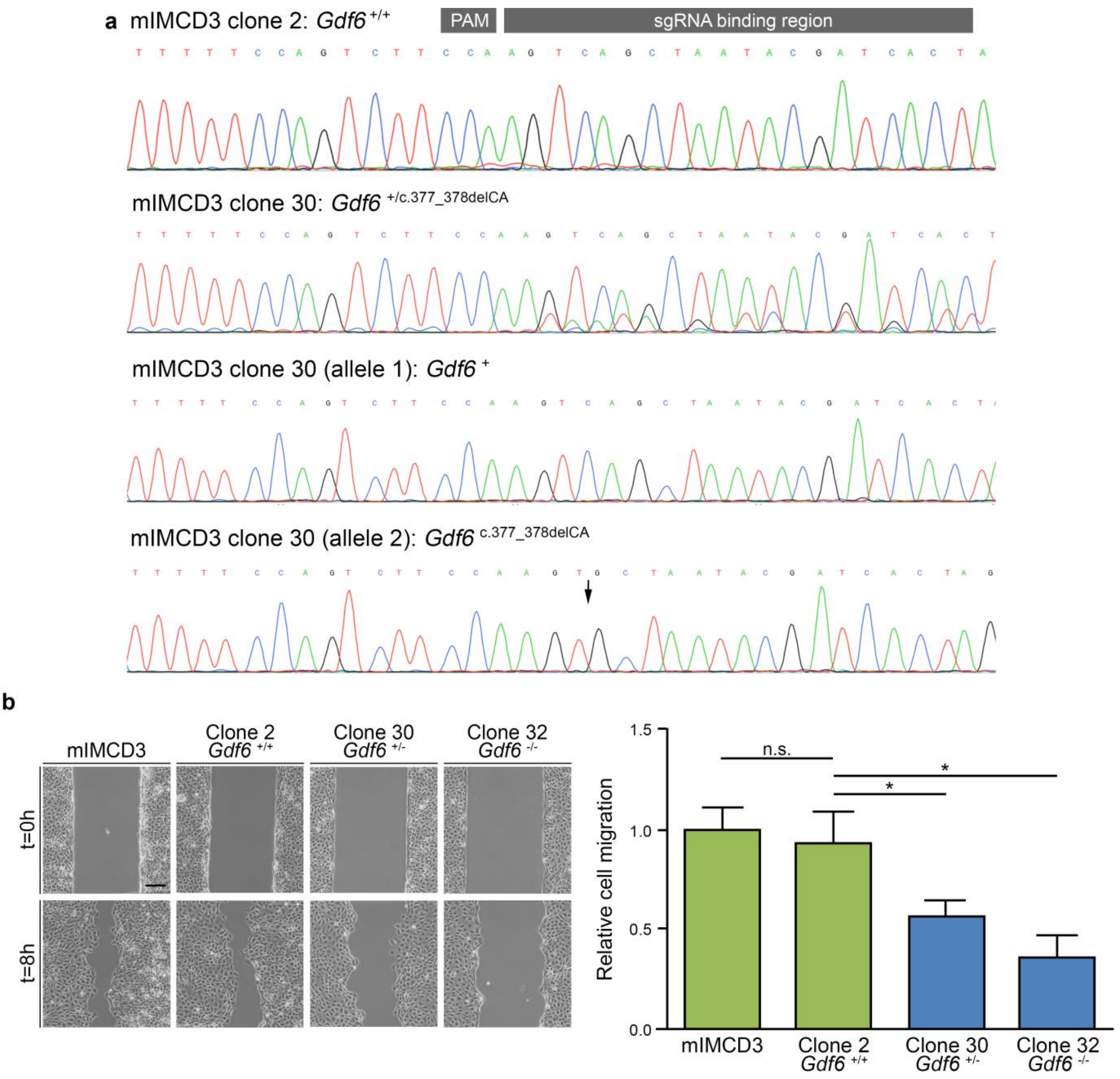

Supplementary Fig. 5 Migration is significantly impaired in *Gdf6*$^{+/-}$ murine IMCD3 cell clone 30 generated by CRISPR-Cas9 technology and analyzed because the *GDF6* variants identified in patients were heterozygous. **a** Electropherograms of the murine *Gdf6* sequence in exon 1 targeted by the single guide RNA (sgRNA) used for CRISPR-Cas9 genome engineering in mIMCD3 cell clone 30 compared to *Gdf6*$^{+/+}$ mIMCD3 cell clone 2. The *Gdf6* sequence variation in mIMCD3 cell clone 30 was heterozygous as confirmed by allele separation revealing the *Gdf6* wildtype sequence on one allele and the deletion c.377_378delCA causing a frameshift predicted to lead to a non-functional protein, p.(Ser126Cysfs*2), or nonsense-mediated mRNA decay on the other allele. The protospacer adjacent motif (PAM) for Cas9 binding and the sgRNA target region are shown in **a**. **b** Migration of *Gdf6*$^{+/-}$ mIMCD3 cell clone 30 was significantly reduced after 8 h compared to *Gdf6*$^{+/+}$ mIMCD3 cell clone 2, but lesser so than migration of *Gdf6*$^{-/-}$ mIMCD3 cell clone 32; results are mean ± SD from three independent experiments. Scale bar: 150 µm.

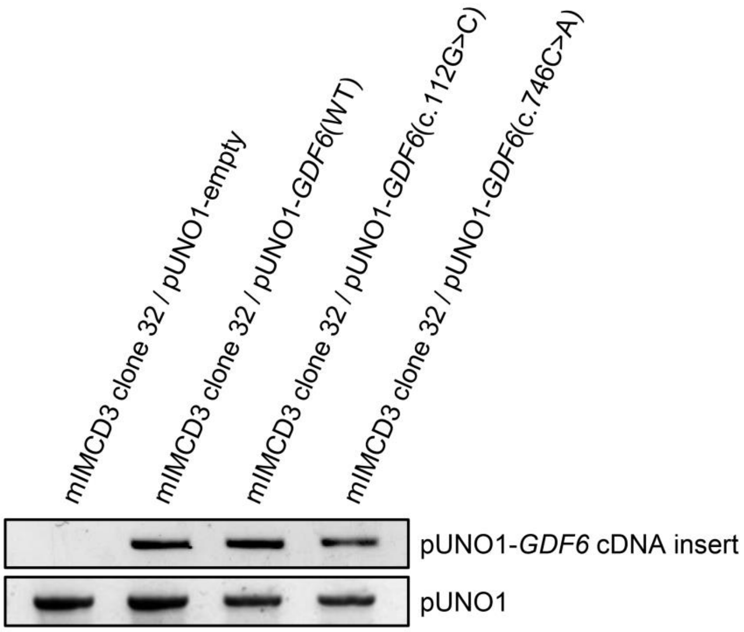

Supplementary Fig. 6 Murine IMCD3 cell clone 32 (*Gdf6*$^{-/-}$) was stably transfected with pUNO1 expression constructs harboring no insert (empty), human *GDF6* wildtype or mutant cDNAs after two weeks of selection using the antibiotic blasticidin S. Primers had been designed to amplify a fragment encompassing sequences of the pUNO1 backbone (pUNO1, bottom row) or of pUNO1 backbone and parts of *GDF6* cDNA insert (pUNO1-*GDF6* cDNA insert, top row) (Supplementary Table 7). PCR amplification was performed using genomic DNA isolated from cells after blasticidin S selection. Stable integration of empty, *GDF6* wildtype- or variant-containing pUNO1 plasmid into genomic DNA was verified in all generated cell lines demonstrating stable transfection.

Supplementary tables

Supplementary Table 1 Renal features of the 194 patients included in this study

Renal anomalies	Frequency
Unilateral renal agenesis	22/194
– with contralateral duplex dysplastic kidney	1/22
– with contralateral duplex kidney	2/22
– with contralateral cystic renal dysplasia	2/22
– with contralateral renal dysplasia	7/22
– with contralateral renal dysplasia and obstructive megaureter	1/22
Unilateral multicystic renal dysplasia	18/194
– with contralateral ureterocele	1/18
– with contralateral duplex dysplastic kidney	1/18
– with contralateral cystic renal dysplasia	1/18
Bilateral cystic renal dysplasia	23/194
– with megacystis	1/23
– with bilateral primary obstructive megaureter	1/23
– with posterior urethral valves	1/23
Unilateral cystic renal dysplasia	2/194
Bilateral renal dysplasia	72/194
– with bilateral pelviureteric junction obstruction	1/72
– with bilateral pelviureteric junction obstruction and bilateral primary obstructive megaureter	1/72
– with bilateral primary obstructive megaureter	1/72
– with megacystis	3/72
– with posterior urethral valves	17/72
– with unilateral pelviureteric junction obstruction	2/72
– with unilateral renal hypoplasia	1/72
Unilateral renal dysplasia	30/194
– with contralateral duplex kidney and hydroureter	1/30
– with posterior urethral valves	1/30
– with unilateral ureterocele	1/30
– with unilateral pelviureteric junction obstruction	1/30
– with renal ectopia	1/30
– with bilateral pelviureteric junction obstruction	1/30
Bilateral renal hypoplasia	2/194
– with posterior urethral valves	1/2
Unilateral renal hypoplasia	11/194
– with bilateral primary obstructive megaureter	1/11
– with renal ectopia	2/11
Bilateral duplex kidney	2/194
– with bilateral ureteroceles	1/2
Unilateral duplex kidney	5/194
– with bilateral pelviureteric junction obstruction	1/5
– with unilateral pelviureteric junction obstruction	1/5
– with contralateral triple kidney, bilateral renal dysplasia	1/5
Horseshoe kidney	5/194
– with primary obstructive megaureter	1/5
Crossed fused renal ectopia	2/194

Number of patients additionally presenting with vesicoureteral reflux: 50/194

Number of patients who had received a kidney transplantation: 31/194

Supplementary Table 2 Analysis of WES data obtained from patient F006.II.1

Filtering steps	Number of variants
Total variants in exomes obtained from whole blood	82,084
Variants with a read depth of ≥15, a call quality of ≥25 and an allele fraction of ≥45%	41,032
Non-silent variants, i.e. splice site (up to 2 bases into intron), frameshift, in-frame indels, stop gained/lost, and non-synonymous missense variants, are retained	6,811
Rare variants (≤1% in the 1000 Genomes Project, ExAC database, gnomAD database, and the NHLBI ESP exomes)[a] are retained	394
Comparison with identically generated exome data of in-house control individuals (n=74), variants not present in controls are retained	185
Variants in genes reported to be mutated in at least one patient with syndromic CAKUT according to our in-house gene list (n=207)[b]	5
Variants predicted to be “deleterious” by at least one prediction tool (MutationTaster, SIFT or PolyPhen-2)[c] are retained	5[d]
Disease causing[e] variant matching the patient’s extrarenal phenotype (Table 1, Supplementary Table 9) is retained and validated by Sanger sequencing	1 (*GDF6*)

[a]1000 Genomes Project data (http://www.internationalgenome.org/), Exome Aggregation Consortium (ExAC; http://exac.broadinstitute.org/), Genome Aggregation Database (gnomAD; http://gnomad.broadinstitute.org/), NHLBI Exome Sequencing Project (http://evs.gs.washington.edu/EVS/)

[b]According to our in-house gene list (adapted from Connaughton et al. 2019; Heidet et al. 2017; Jain and Chen 2019; Kosfeld et al. 2018; Nicolaou et al. 2016; van der Ven et al. 2018a,b; Vivante and Hildebrandt 2016) comprising the following genes: *ACTB, ACTG1, AHI1, AIFM3, AMER1, ARID1B, ARL6, ATP7A, ATXN10, AXIN1, B3GALTL, BBS1, BBS2, BBS4, BBS5, BBS6, BBS7, BBS8, BBS9, BBS10, BBS12, BCOR, BICC1, BMP7, BRAF, BSCL2, CD96, CD151, CDC5L, CDKN1C, CEP290, CHD7, CHRNG, CISD2, CREBBP, CTU2, CYP21, DACH1, DACT1, DHCR7, DIS3L2, DLG3, DYNC2H1, EMG1, EP300, ERCC8, ESCO2, ESRRG, ETFA, ETFB, ETFDH, EVC, EVC2, EYA1, FAM58A, FANCA, FANCB, FANCD2, FANCE, FANCI, FANCL, FAT4, FBN1, FGF3, FGF8, FGF10, FGFR1, FGFR2, FGFR3, FLNA, FMN1, FOXC1, FOXF1, FOXP1, FRAS1, GDF3, GDF6, GDF11, GDNF, GFRA1, GLI2, GLI3, GLIS2, GPC3, H19, HES7, HOXA13, HOXD13, HPSE2, HYLS1, ICK, IFT27, IFT46, IFT52, IFT57, IFT74, IFT80, IFT81, IFT172, INPP5E, INVS, ITGA3, JAG1, JAM3, KAT6B, KCNH2, KCNQ10T1, KCTD1, KMT2D, KRAS, LFNG, LMNA, LMX1B, LPP, LRIG2, LRP2, LRP4, MAP2K1, MAP2K2, MESP2, MID1, MKKS, MKS1, MKS3, MYCN, NEK1, NEK8, NFIX, NIPBL, NOTCH2, NPHP1, NPHP3, NPHP4, NSDHL, OFD1, OSR1, PAX2, PAX8, PCSK5, PEX1, PEX5, PIGA, PIGL, PIGN, PIGO, PIGT, PIGV, PIGY, PKD1, PKD2, PKHD1, PMM2, POC1A, PORCN, PROK2, PROKR2, PTEN, PTF1A, PTPN11, RAF1, RAI1, RECQL4, ROR2, RPGRIP1L, RPS19, RPS24, SALL1, SALL4, SCARF2, SDCCAG8, SEMA3A, SEMA3E, SETBP1, SF3B4, SH2B1, SHH, SMC1A, SNAP29, SOS1, SOX9, SRCAP, STRA6, TBX1, TBX3, TFAP2A, TMCO1, TMEM67, TMEM216, TMEM231, TP63, TRIM32, TRPS1, TSC1, TSC2, TTC21B, TTC30A, TWIST2, UBE3A, UBR1, UPF3B, VANGL1, WFS1, WNT3, WNT4, WNT5A, ZIC3, ZMPSTE24*

[c]MutationTaster (http://www.mutationtaster.org), SIFT (http://sift.jcvi.org/), PolyPhen-2 (http://genetics.bwh.harvard.edu/pph2/)

[d]Variants are summarized in Supplementary Table 3

[e]According to HGMD Professional (https://www.qiagenbioinformatics.com/products/human-gene-mutation-database/)

Supplementary Table 3 WES data obtained from patient F006.II.1: five non-silent rare variants in genes associated with human syndromic CAKUT (n=207) not found in in-house controls and predicted to be disease causing. See separate .xlsx-file.

Supplementary Table 4 Oligonucleotides used for amplification and sequencing of human *GDF6* coding exon

Exon	Oligonucleotide name	Sequence (5´ to 3´)
1	*GDF6*-E1F	TGCCCTCCCTCCCCATTC
	GDF6-E1R	CCGCAGCCACATTCAGAAAC
2	*GDF6*-E2F1	GAGCACCGGGCTGATCTC
	GDF6-E2R1	CAGCCAAGGCCTGGCATC
	GDF6-E2F2	GTATTCACCAGATCCCAGCG
	GDF6-E2R2	CTTCCTCCTCCGCCTCTC

Supplementary Table 5 Oligonucleotides used for amplification and sequencing of murine *Gdf6* exon 1 including the CRISPR/Cas9 target site

Exon	Oligonucleotide name	Sequence (5´ to 3´)
1	*Gdf6*-E1F	TATGGATCCATGGACACTCCTAGG
	Gdf6-E1R	ATAGAATTCGCCTGGGTAGAGCTC

Supplementary Table 6 Oligonucleotides used for cloning of human *GDF6* expression constructs

Vector	Oligonucleotide name	Sequence (5´ to 3´)
pUNO1	*GDF6*-pUNO1-F	GCGTGTCGACGGATCCATGGATACTCCCAGGGTCCTG
	GDF6-pUNO1-R	TGTCTGGCCAGCTAGCCTACCTGCAGCCGCACGA

Supplementary Table 7 Oligonucleotides used for site-directed mutagenesis of pUNO1-*GDF6* expression constructs

Vector	Oligonucleotide name	Sequence (5´ to 3´)
pUNO1	*GDF6*-c.112G>C-F	[PHO]AGCTGCGTTCCACCAAGGG
	GDF6-c.112G>C-R	CGGCGGACGACGAGGA
	GDF6-c.746C>A-F	[PHO]CGCGCGAGCGGGGACCCCAGCAACCGCCGC
	GDF6-c.746C>A-R	CCTCGGCCTCCCCGGCGTCCAGCTCGCCCC

Supplementary Table 8 Oligonucleotides used for analysis of stable transfection of mIMCD3 cells

Amplicon name	Oligonucleotide name	Sequence (5´ to 3´)
pUNO1-*GDF6* cDNA insert	pUNO1-*GDF6*-F	TCACTGCATTCTAGTTGTGG
	pUNO1-*GDF6*-R	CCAGTCTTCCAAGTCGGCTA
pUNO1	pUNO1-F	CCACAACTAGAATGCAGTGA
	pUNO1-R	CAAATGGGCGGTAGGCGTG

Supplementary Table 9 Clinical features of 86 individuals carrying a variant in the *GDF6* gene reported in this or previous studies

Reference	No. of variant carriers[1]	Nucleotide change	Amino acid change	Renal anomalies	Skeletal anomalies	Ocular anomalies	Auricular anomalies	Other anomalies
Retterer et al. 2016	1	c.73C>T	p.(Gln25*)	Multiple congenital anomalies				
This study	1	c.112G>C	p.(Gly38Arg)	Kidney hypodysplasia (n=1)	Macrocephaly, high-arched palate (n=1)	Short narrow palpebral fissures (n=1)	None	None
Asai-Coakwell et al. 2009	1	c.125G>T	p.(Gly42Val)	None	Spondylothoracic dysostosis (n=1)	None	None	None
Huang et al. 2015	1	c.136C>T	p.(Arg46Cys)	Not specified	Not specified	Primary angle-closure glaucoma (n=1)	Not specified	Not specified
Asai-Coakwell et al. 2013	2	c.169G>C	p.(Asp57His)	Not specified	Not specified	Leber congenital amaurosis (n=1)	Not specified	Not specified
Asai-Coakwell et al. 2009	1	c.356A>G	p.(Gln119Arg)	None	None	Microphthalmia (n=1)	None	None
Gonzalez-Rodriguez et al. 2010	1	c.460A>C	p.(Met154Thr)	None	None	Bilateral anophthalmia (n=1)	None	Phimosis (n=1)
Ye et al. 2010	1	c.595G>A	p.(Ala199Thr)	Not specified	None	Bilateral coloboma, microphthalmia, nystagmus (n=1)	Not specified	Not specified
Asai-Coakwell et al. 2013	2	c.595G>A	p.(Ala199Thr)	Not specified	Not specified	Leber congenital amaurosis or juvenile retinitis pigmentosa (n=1)	Not specified	Not specified
Asai-Coakwell et al. 2009	1	c.647A>G	p.(Asp216Gly)	None	None	Microphthalmia (n=1)	None	None
This study	7	c.746C>A	p.(Ala249Glu)	Crossed fused renal ectopia, hydronephrosis, megaureters, VUR (n=1), kidney hypodysplasia (n=1)	Scoliosis (n=2), fused, butterfly or missing vertebral bodies (n=1), high-arched palate (n=2), prognathism (n=1), short stature (n=1)	Anisometropia with hyperopia, astigmatism, amblyopia, suspected microphthalmia, corneal opacities (n=1)	Auricle dysplasia, aplasia of the external auditory canal (n=1), preauricular pits (n=2), lop ears (2)	Low standing conus medullaris, anal atresia, rectovestibular fistula, small ventricular septal defects, patent foramen ovale (n=1), short frenulum (n=1)
Tassabehji et al. 2008	6	c.746C>A	p.(Ala249Glu)	None	Vertebral fusions (n=6), flaring of the lower ribs (n=2), negative ulna variance	None	None	Intervertebral disc degeneration (n=1), omphalocele (n=1)
Asai-Coakwell et al. 2009	3	c.746C>A	p.(Ala249Glu)	None	Klippel-Feil (n=1), post-axial polydactyly (n=1)	Microphthalmia (n=1), coloboma (n=1)	None	None
den Hollander et al. 2010	4	c.746C>A	p.(Ala249Glu)	None	Cleft palate, hemivertebrae, talipes (n=1)	Microphthalmia (n=3)	Malformed ossicles (n=1)	Hydrocephalus, small pituitary gland, growth hormone deficiency (n=1)

[1]Including carrier siblings and parents, if applicable and data were provided

Supplementary Table 9 continued

Reference	No. of variant carriers[1]	Nucleotide change	Amino acid change	Renal anomalies	Skeletal anomalies	Ocular anomalies	Auricular anomalies	Other anomalies
Gonzalez-Rodriguez et al. 2010	3	c.746C>A	p.(Ala249Glu)	None	Cleft palate (n=1)	Bilateral microphthalmia (n=2), bilateral nanophthalmia (n=1)	None	Cleft lip (n=1)
Asai-Coakwell et al. 2013	5			Not specified	Not specified	Leber congenital amaurosis (n=1), Leber congenital amaurosis or juvenile retinitis pigmentosa (n=1)	Not specified	Not specified
Markunas et al. 2013	4			None	Chiari type I malformation (n=3), suspected Chiari type 0 malformation (n=1)	None	None	Syringomyelia
Slavotinek et al. 2015	1			Not specified	Not specified	Bilateral anophthalmia (n=1)	Not specified	Not specified
Asai-Coakwell et al. 2009	1	c.758A>T	p.(Gln253Leu)	None	None	Microphthalmia (n=1)	None	Single testis (n=1)
Tassabehji et al. 2008	2	c.866T>C	p.(Leu289Pro)	Absent right kidney (n=1)	Macrocephaly, Sprengel anomaly of the left shoulder (n=1), Chiari type II malformation, multiple segmentation anomalies affecting the entire spine and ribs, hemi- and butterfly-vertebrae, fifth finger clinodactyly, rocker-bottom feet (n=1)	Strabismus (n=1)	None	Short neck, mirror movements (n=1), dilatation of the lateral ventricles, absent uterus, anal atresia (n=1)
Asai-Coakwell et al. 2013	2	c.876G>A	p.(Glu292Asp)	Not specified	Not specified	Leber congenital amaurosis or juvenile retinitis pigmentosa (n=1)	Not specified	Not specified
Asai-Coakwell et al. 2009	1	c.980C>A	p.(Pro327His)	None	None	Microphthalmia (n=1)	None	None
Chassaing et al. 2014	2			None	None	Micro coloboma (n=1)	None	None
Drage Berentsen et al. 2019	6	c.1197C>A	p.(Asn399Lys)	None	Multiple synostoses syndrome including carpal and tarsal synostoses (n=6)	None	None	None
Asai-Coakwell et al. 2009	1	c.1271A>G	p.(Lys424Arg)	Fused (horseshoe) kidney (n=1)	Hemi-vertebrae, rib malformation (n=1)	None	None	None
Huang et al. 2015	1			Not specified	Not specified	Primary open-angle glaucoma (n=1)	Not specified	Not specified
Terhal et al. 2018	6	c.1287C>A	p.(Ser429Arg)	Not specified	Multiple synostoses syndrome including carpal and tarsal synostoses (n=5)	None	Hearing loss (n=1), otosclerosis (n=2)	Not specified
Huang et al. 2015	1	c.1288A>G	p.(Ile430Val)	Not specified	Not specified	Primary angle-closure glaucoma (n=1)	Not specified	Not specified
Wang et al. 2016	18	c.1330T>A	p.(Tyr444Asn)	Not specified	Multiple synostoses syndrome (n=18), feet deformation (n=23)	Not specified	Hearing difficulty (n=15)	Hypertension (n=1), diabetes (n=3)

[1]Including carrier siblings and parents, if applicable and data were provided

Supplementary references

Asai-Coakwell M, French CR, Ye M, Garcha K, Bigot K, Perera AG, Staehling-Hampton K, Mema SC, Chanda B, Mushegian A, Bamforth S, Doschak MR, Li G, Dobbs MB, Giampietro PF, Brooks BP, Vijayalakshmi P, Sauve Y, Abitbol M, Sundaresan P, van Heyningen V, Pourquie O, Underhill TM, Waskiewicz AJ, Lehmann OJ (2009) Incomplete penetrance and phenotypic variability characterize Gdf6-attributable oculo-skeletal phenotypes. Hum Mol Genet 18:1110–1121. https://doi.org/10.1093/hmg/ddp008

Asai-Coakwell M, March L, Dai XH, Duval M, Lopez I, French CR, Famulski J, Baere Ed, Francis PJ, Sundaresan P, Sauve Y, Koenekoop RK, Berry FB, Allison WT, Waskiewicz AJ, Lehmann OJ (2013) Contribution of growth differentiation factor 6-dependent cell survival to early-onset retinal dystrophies. Hum Mol Genet 22:1432–1442. https://doi.org/10.1093/hmg/dds560

Chassaing N, Causse A, Vigouroux A, Delahaye A, Alessandri J-L, Boespflug-Tanguy O, Boute-Benejean O, Dollfus H, Duban-Bedu B, Gilbert-Dussardier B, Giuliano F, Gonzales M, Holder-Espinasse M, Isidor B, Jacquemont M-L, Lacombe D, Martin-Coignard D, Mathieu-Dramard M, Odent S, Picone O, Pinson L, Quelin C, Sigaudy S, Toutain A, Thauvin-Robinet C, Kaplan J, Calvas P (2014) Molecular findings and clinical data in a cohort of 150 patients with anophthalmia/microphthalmia. Clin Genet 86:326–334. https://doi.org/10.1111/cge.12275

Connaughton DM, Kennedy C, Shril S, Mann N, Murray SL, Williams PA, Conlon E, Nakayama M, van der Ven AT, Ityel H, Kause F, Kolvenbach CM, Dai R, Vivante A, Braun DA, Schneider R, Kitzler TM, Moloney B, Moran CP, Smyth JS, Kennedy A, Benson K, Stapleton C, Denton M, Magee C, O'Seaghdha CM, Plant WD, Griffin MD, Awan A, Sweeney C, Mane SM, Lifton RP, Griffin B, Leavey S, Casserly L, de Freitas DG, Holian J, Dorman A, Doyle B, Lavin PJ, Little MA, Conlon PJ, Hildebrandt F (2019) Monogenic causes of chronic kidney disease in adults. Kidney Int 95:914–928. https://doi.org/10.1016/j.kint.2018.10.031

den Hollander AI, Biyanwila J, Kovach P, Bardakjian T, Traboulsi EI, Ragge NK, Schneider A, Malicki J (2010) Genetic defects of GDF6 in the zebrafish out of sight mutant and in human eye developmental anomalies. BMC Genet 11:102. https://doi.org/10.1186/1471-2156-11-102

Drage Berentsen R, Haukanes BI, Juliusson PB, Rosendahl K, Houge G (2019) A Novel GDF6 Mutation in a Family with Multiple Synostoses Syndrome without Hearing Loss. Mol Syndromol 9: 228–234. https://doi.org/10.1159/000492418

Gonzalez-Rodriguez J, Pelcastre EL, Tovilla-Canales JL, Garcia-Ortiz JE, Amato-Almanza M, Villanueva-Mendoza C, Espinosa-Mattar Z, Zenteno JC (2010) Mutational screening of CHX10, GDF6, OTX2, RAX and SOX2 genes in 50 unrelated microphthalmia-anophthalmia-coloboma (MAC) spectrum cases. Br J Ophthalmol 94:1100–1104. https://doi.org/10.1136/bjo.2009.173500

Heidet L, Moriniere V, Henry C, De Tomasi L, Reilly ML, Humbert C, Alibeu O, Fourrage C, Bole-Feysot C, Nitschke P, Tores F, Bras M, Jeanpierre M, Pietrement C, Gaillard D, Gonzales M, Novo R, Schaefer E, Roume J, Martinovic J, Malan V, Salomon R, Saunier S, Antignac C, Jeanpierre C (2017) Targeted Exome Sequencing Identifies PBX1 as Involved in Monogenic Congenital Anomalies of the Kidney and Urinary Tract. J Am Soc Nephrol 28:2901–2914. https://doi.org/10.1681/ASN.2017010043

Huang X, Xiao X, Jia X, Li S, Li M, Guo X, Liu X, Zhang Q (2015) Mutation analysis of the genes associated with anterior segment dysgenesis, microcornea and microphthalmia in 257 patients with glaucoma. Int J Mol Med 36:1111–1117. https://doi.org/10.3892/ijmm.2015.2325

Jain S, Chen F (2019) Developmental pathology of congenital kidney and urinary tract anomalies. Clin Kidney J 12:382–399. https://doi.org/10.1093/ckj/sfy112

Kosfeld A, Martens H, Hennies I, Haffner D, Weber RG (2018) Kongenitale Anomalien der Nieren und ableitenden Harnwege (CAKUT). med gen 30:448–460. https://doi.org/10.1007/s11825-018-0226-y

Markunas CA, Soldano K, Dunlap K, Cope H, Asiimwe E, Stajich J, Enterline D, Grant G, Fuchs H, Gregory SG, Ashley-Koch AE (2013) Stratified whole genome linkage analysis of Chiari type I malformation implicates known Klippel-Feil syndrome genes as putative disease candidates. PLoS ONE 8:e61521. https://doi.org/10.1371/journal.pone.0061521

Moorman AF, Houweling AC, Boer PAd, Christoffels VM (2001) Sensitive nonradioactive detection of mRNA in tissue sections: Novel application of the whole-mount in situ hybridization protocol. J Histochem Cytochem 49:1–8. https://doi.org/10.1177/002215540104900101

Nicolaou N, Pulit SL, Nijman IJ, Monroe GR, Feitz WF, Schreuder MF, van Eerde AM, de Jong TP, Giltay JC, van der Zwaag B, Havenith MR, Zwakenberg S, van der Zanden LF, Poelmans G, Cornelissen EA, Lilien MR, Franke B, Roeleveld N, van Rooij IA, Cuppen E, Bongers EM, Giles RH, Knoers NV, Renkema KY (2016) Prioritization and burden analysis of rare variants in 208 candidate genes suggest they do not play a major role in CAKUT. Kidney Int 89:476–486. https://doi.org/10.1038/ki.2015.319

Ran FA, Hsu PD, Wright J, Agarwala V, Scott DA, Zhang F (2013) Genome engineering using the CRISPR-Cas9 system. Nat Protoc 8:2281–2308. https://doi.org/10.1038/nprot.2013.143

Retterer K, Juusola J, Cho MT, Vitazka P, Millan F, Gibellini F, Vertino-Bell A, Smaoui N, Neidich J, Monaghan KG, McKnight D, Bai R, Suchy S, Friedman B, Tahiliani J, Pineda-Alvarez D, Richard G, Brandt T, Haverfield E, Chung WK, Bale S (2016) Clinical application of whole-exome sequencing across clinical indications. Genet Med 18:696–704. https://doi.org/10.1038/gim.2015.148

Schille C, Bayerlova M, Bleckmann A, Schambony A (2016) Ror2 signaling is required for local upregulation of GDF6 and activation of BMP signaling at the neural plate border. Development 143:3182–3194. https://doi.org/10.1242/dev.135426

Schindelin J, Arganda-Carreras I, Frise E, Kaynig V, Longair M, Pietzsch T, Preibisch S, Rueden C, Saalfeld S, Schmid B, Tinevez JY, White DJ, Hartenstein V, Eliceiri K, Tomancak P, Cardona A (2012) Fiji: an open-source platform for biological-image analysis. Nat Methods 9:676–682. https://doi.org/10.1038/nmeth.2019

Schneider CA, Rasband WS, Eliceiri KW (2012) NIH Image to ImageJ: 25 years of image analysis. Nat Methods 9:671–675. https://doi.org/10.1038/nmeth.2089

Sive HL, Grainger R, Harland RM (2000) Early development of Xenopus laevis: a laboratory manual. Cold Spring Harbor Laboratory Press, Cold Spring Harbor, N.Y., USA.

Slavotinek AM, Garcia ST, Chandratillake G, Bardakjian T, Ullah E, Wu D, Umeda K, Lao R, Tang PL-F, Wan E, Madireddy L, Lyalina S, Mendelsohn BA, Dugan S, Tirch J, Tischler R, Harris J, Clark MJ, Chervitz S, Patwardhan A, West JM, Ursell P, Alba Campomanes Ad, Schneider A, Kwok P-Y, Baranzini S, Chen RO (2015) Exome sequencing in 32 patients with anophthalmia/microphthalmia and developmental eye defects. Clin Genet 88:468–473. https://doi.org/10.1111/cge.12543

Tassabehji M, Fang ZM, Hilton EN, McGaughran J, Zhao Z, Bock CEd, Howard E, Malass M, Donnai D, Diwan A, Manson FDC, Murrell D, Clarke RA (2008) Mutations in GDF6 are associated with vertebral segmentation defects in Klippel-Feil syndrome. Hum Mutat 29:1017–1027. https://doi.org/10.1002/humu.20741

Terhal PA, Verbeek NE, Knoers N, Nievelstein RJAJ, van den Ouweland A, Sakkers RJ, Speleman L, van Haaften G (2018) Further delineation of the GDF6 related multiple synostoses syndrome. Am J Med Genet A 176:225–229. https://doi.org/10.1002/ajmg.a.38503

van der Ven AT, Vivante A, Hildebrandt F (2018a) Novel Insights into the Pathogenesis of Monogenic Congenital Anomalies of the Kidney and Urinary Tract. J Am Soc Nephrol 29:36–50. https://doi.org/10.1681/ASN.2017050561

van der Ven AT, Connaughton DM, Ityel H, Mann N, Nakayama M, Chen J, Vivante A, Hwang DY, Schulz J, Braun DA, Schmidt JM, Schapiro D, Schneider R, Warejko JK, Daga A, Majmundar AJ, Tan W, Jobst-Schwan T, Hermle T, Widmeier E, Ashraf S, Amar A, Hoogstraaten CA, Hugo H, Kitzler TM, Kause F, Kolvenbach CM, Dai R, Spaneas L, Amann K, Stein DR, Baum MA, Somers MJG, Rodig NM, Ferguson MA, Traum AZ, Daouk GH, Bogdanovic R, Stajic N, Soliman NA, Kari JA, El Desoky S, Fathy HM, Milosevic D, Al-Saffar M, Awad HS, Eid LA, Selvin A, Senguttuvan P, Sanna-Cherchi S, Rehm HL, MacArthur DG, Lek M, Laricchia KM, Wilson MW, Mane SM, Lifton RP, Lee RS, Bauer SB, Lu W, Reutter HM, Tasic V, Shril S, Hildebrandt F (2018b) Whole-Exome Sequencing Identifies Causative Mutations in Families with Congenital Anomalies of the Kidney and Urinary Tract. J Am Soc Nephrol 29:2348–2361. https://doi.org/10.1681/ASN.2017121265

Vivante A, Hildebrandt F (2016) Exploring the genetic basis of early-onset chronic kidney disease. Nat Rev Nephrol 12:133–146. https://doi.org/10.1038/nrneph.2015.205

Wang J, Yu T, Wang Z, Ohte S, Yao R-E, Zheng Z, Geng J, Cai H, Ge Y, Li Y, Xu Y, Zhang Q, Gusella JF, Fu Q, Pregizer S, Rosen V, Shen Y (2016) A New Subtype of Multiple Synostoses Syndrome Is Caused by a Mutation in GDF6 That Decreases Its Sensitivity to Noggin and Enhances Its Potency as a BMP Signal. J Bone Miner Res 31:882–889. https://doi.org/10.1002/jbmr.2761

Ye M, Berry-Wynne KM, Asai-Coakwell M, Sundaresan P, Footz T, French CR, Abitbol M, Fleisch VC, Corbett N, Allison WT, Drummond G, Walter MA, Underhill TM, Waskiewicz AJ, Lehmann OJ (2010) Mutation of the bone morphogenetic protein GDF3 causes ocular and skeletal anomalies. Hum Mol Genet 19:287–298. https://doi.org/10.1093/hmg/ddp496
